# Age-dependent pathogenic characteristics of SARS-CoV-2 infection in ferrets

**DOI:** 10.1038/s41467-021-27717-3

**Published:** 2022-01-10

**Authors:** Young-Il Kim, Kwang-Min Yu, June-Young Koh, Eun-Ha Kim, Se-Mi Kim, Eun Ji Kim, Mark Anthony B. Casel, Rare Rollon, Seung-Gyu Jang, Min-Suk Song, Su-Jin Park, Hye Won Jeong, Eung-Gook Kim, Ok-Jun Lee, Yong-Dae Kim, Younho Choi, Shin-Ae Lee, Youn Jung Choi, Su-Hyung Park, Jae U. Jung, Young Ki Choi

**Affiliations:** 1 College of Medicine and Medical Research Institute, Chungbuk National University, Cheongju, Republic of Korea; 2 Zoonotic Infectious Diseases Research Center, Chungbuk National University, Cheongju, Korea; 3 Center for Study of Emerging and Re-emerging Viruses, Korea Virus Research Institute, Institute for Basic Science (IBS), Daejeon, 34126 Republic of Korea; 4 Graduate School of Medical Science and Engineering, Korea Advanced Institute of Science and Technology (KAIST), Daejeon, Republic of Korea; 5 Division of Life Science, Research Institute of Life Science, Gyeongsang National University, Jinju, 52828 Korea; 6 Cancer Biology Department and Global Center for Pathogens Research and Human Health, Lerner Research Institute, Cleveland Clinic, Cleveland, OH USA

**Keywords:** Viral host response, Viral transmission, SARS-CoV-2

## Abstract

While the seroprevalence of SARS-CoV-2 in healthy people does not differ significantly among age groups, those aged 65 years or older exhibit strikingly higher COVID-19 mortality compared to younger individuals. To further understand differing COVID-19 manifestations in patients of different ages, three age groups of ferrets are infected with SARS-CoV-2. Although SARS-CoV-2 is isolated from all ferrets regardless of age, aged ferrets (≥3 years old) show higher viral loads, longer nasal virus shedding, and more severe lung inflammatory cell infiltration, and clinical symptoms compared to juvenile (≤6 months) and young adult (1–2 years) groups. Furthermore, direct contact ferrets co-housed with the virus-infected aged group shed more virus than direct-contact ferrets co-housed with virus-infected juvenile or young adult ferrets. Transcriptome analysis of aged ferret lungs reveals strong enrichment of gene sets related to type I interferon, activated T cells, and M1 macrophage responses, mimicking the gene expression profile of severe COVID-19 patients. Thus, SARS-CoV-2-infected aged ferrets highly recapitulate COVID-19 patients with severe symptoms and are useful for understanding age-associated infection, transmission, and pathogenesis of SARS-CoV-2.

## Abstract (web summary)

Here, Kim et al. characterize SARS-CoV-2 infection in juvenile, young, and old aged ferrets to provide a further understanding of differences in COVID-19 severity in humans at different ages. Aged ferrets have higher viral loads, shed virus longer, and mimic the transcriptomic profile of severely infected patients.

## Introduction

Coronavirus Disease 2019 (COVID-19) is caused by a novel emerging Severe Acute Respiratory Syndrome Coronavirus 2 (SARS-CoV-2)^1^. Since the first outbreak in China in November 2019, COVID-19 has spread rapidly and globally with a mortality rate of ∼2%, resulting in a serious global health crisis. Thus, the World Health Organization (WHO) declared COVID-19 a pandemic on March 11, 2020^2^. SARS-CoV-2 is the third identified highly pathogenic coronavirus transmitted from animals to humans since the first outbreak of SARS-CoV-1^3^ in 2002 and Middle East Respiratory Syndrome Coronavirus (MERS-CoV)^4^ in 2012. However, the magnitude of the impact of SARS-CoV-2 is by far the greatest due to the significantly larger number of human cases. Consequently, this is the first coronavirus-associated pandemic with a marked number of human deaths.

Despite strict precautionary measures, such as social distancing policies and restriction of social gatherings, the number of SARS-CoV-2 infections continues to grow exponentially with a proportional increase in deaths^5^. Fortunately, several licensed COVID-19 vaccines with high safety and efficacy have been developed in record time and are expected to reach all populations by the end of this year. However, risk factors for COVID-19-associated deaths include age, sex, and comorbidities of affected individuals. Thus, it is imperative to understand the etiology of severe disease and determine the most effective treatment strategies for high-risk groups. In particular, increased age is the greatest risk factor for severe COVID-19, and although severe COVID-19 has been reported among all age groups, 90% of patients with severe COVID-19 are above 30 years of age^6^. Moreover, higher morbidity and mortality rates have consistently been observed in aged human populations throughout the COVID-19 pandemic^7^. When compared to the young adult population, the aged population is generally more susceptible to respiratory infections and show poor prognoses^8^. Similarly, mouse-adapted SARS-CoV-2 showed strong age-dependent clinical phenotypes in a mouse model^9,10^. Aged mice were found to exhibit signs of severe lung damage^11–14^ and higher viral replication with longer periods of viral shedding. Furthermore, several age-dependent pathogenesis studies using hamsters^15–17^ and non-human primates^18–20^ have also been reported. Aged hamsters develop severe disease manifestations and some animals succumb to SARS-CoV-2 infection. Consistently, studies in non-human primates showed that aged monkeys exhibit more severe disease phenotypes than their younger counterparts. Furthermore, in several previous studies clinical symptoms of SARS-CoV-2 infection were observed in ferrets, despite variations in the age of the ferrets and virus strain.

In addition to the various clinical symptoms reported for COVID-19, systematic experimental studies are needed to further dissect the diverse disease manifestations among different age groups. While human clinical studies are highly valuable, a number of limitations including ethical issues, behavioral and environmental variables, and medical history of the patients, may impede identification of the fundamental cause of the disease in a timely manner. Hence, this necessitates the development of an appropriate animal model to aid in understanding transmission and pathogenesis of SARS-CoV-2, as well as elucidating host immune responses against SARS-CoV-2 infection. The current hACE2 transgenic mouse model, which expresses human ACE2, the entry receptor for SARS-CoV-2, showed weight loss and virus replication in the lung following SARS-CoV-2 infection. Although some mice showed neurological symptoms which led to fatal infection in a virus dose-dependent manner^13,21^, other clinical symptoms of infection, such as body temperature, sneezing, coughing, and lethargy, are difficult to monitor in the mouse model. Furthermore, neurologic-related mortality also brings the suitability of this animal model into debate, as infection of the central nervous system is rarely observed in COVID-19 patients^22,23^, underscoring the need for an animal model that better represents human COVID-19 manifestation. Following the fortuitous discovery that ferrets are susceptible to human influenza viruses, ferrets have been recognized as a useful animal model for the study of respiratory viruses, such as respiratory syncytial virus, parainfluenza viruses, and SARS coronavirus^24–27^. Ferrets have a respiratory tract histo-anatomically analogous to that of humans, with similar anatomic proportions of the upper and lower respiratory tracts, density of submucosal glands in the bronchial wall, and number of generations of terminal bronchioles^26^, further supporting the adequacy of this model for study of human respiratory viral infections.

To address pressing scientific questions ranging from basic virology to the development and assessment of novel drugs and vaccines for COVID-19, we have recently established a ferret model for SARS-CoV-2 infection and transmission that highly recapitulates pathological aspects of the human infection^28^. SARS-CoV-2-infected ferrets exhibited elevated body temperatures and virus replication; infected ferrets shed the virus through nasal washes and saliva, urine, and fecal specimens; SARS-CoV-2 was readily transmitted to naïve direct-contact ferrets, but less efficiently to naive indirect-contact ferrets, and acute bronchiolitis was observed in infected lungs^28^. Thus, SARS-CoV-2 replicates efficiently in the respiratory tracts of ferrets without prior adaption, and investigation of viral transmission in SARS-CoV-2 infected ferrets revealed the ability to spread to naïve ferrets both through direct contact and indirectly through respiratory droplets^28–30^. Unlike mice and hamsters^13,16^, ferrets exhibit a clinical presentation that closely resemble human symptoms (coughing, nasal discharge, and fatigue) of COVID-19^28,31,32^. Also, we have recently developed an aged ferret infection model (≥4 years old, equivalent to 70 years old in humans) for the emerging human severe fever with thrombocytopenia syndrome virus (SFTSV) that fully recapitulates human clinical manifestation^33^. SFTSV infection exhibits severe clinical manifestation and increased fatality rate in patients 50 years and older, which was recapitulated in SFTSV-infected aged ferrets, as evidenced by disease symptoms such as high temperature, weight loss, severe thrombocytopenia, and death.

In this study, we demonstrate the age-related disease severity observed in COVID-19 patients by performing SARS-CoV-2 infection in ferrets of three different age groups: ferrets under 6 months to simulate juveniles/children (G1), one to two-year-old to simulate young adults (G2), and 3 years or older to simulate patients over 50 years old (G3). We compare disease severity among the three groups by examining clinical symptoms, viral load in the respiratory tract, and lung histopathology. Furthermore, RNA sequencing analysis with lung tissues from SARS-CoV-2-infected young adult or aged ferrets reveals differences in global and dynamic gene expression. As a result, the aged (G3) ferret group shows a higher viral load and more severe clinical symptoms than the juvenile (G1) and young adult (G2) ferret groups. Furthermore, contact ferrets co-housed with infected aged ferrets sheds significantly high viral titers through their respiratory tracts and exhibits high clinical disease scores. In addition, although an IgG antibody response to SARS-CoV-2 is not induced in the juvenile group, the young adult and aged contact groups show detectable IgG in an age-dependent manner. Transcriptome analysis of the aged ferret group reveals the highest expression of gene sets related to type I interferon (IFN), activated T cells, and M1 macrophage responses. Thus, SARS-CoV-2-infected aged ferrets considerably resemble aged COVID-19 patients with severe clinical symptoms^34^. This study demonstrates that the aged ferret model manifests age-dependent etiology of severe COVID-19, indicating feasibility for studies in the development of tailored therapeutics and vaccines for the specific target group.

## Results

### Clinical features of SARS-CoV-2 infection among ferrets of different ages

**Fig. 1:**
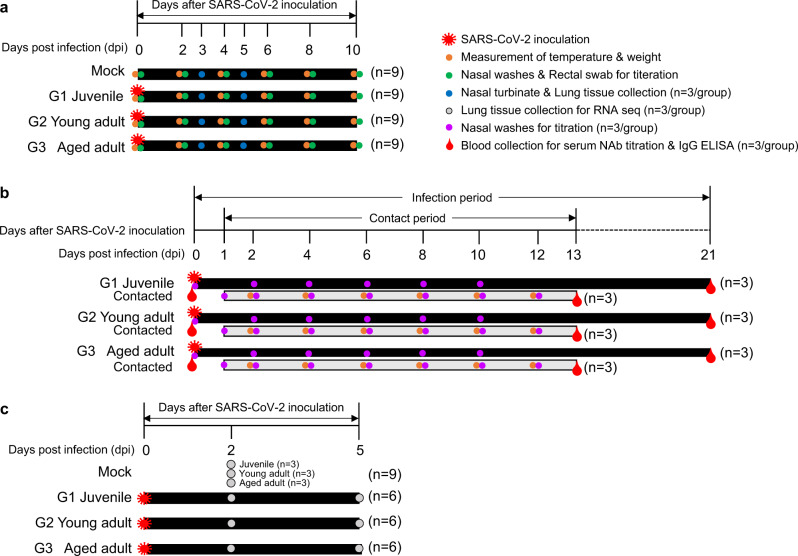
Schematic diagrams illustrating ferret study design. Experimental layout of age-dependent pathogenesis of SARS-CoV-2 in ferret animal model. **a** Timetable showing time points of virus inoculation, observation and monitoring of clinical manifestation and sample collection in infected ferrets by age groups. Orange circle denotes for time points of temperature and weight measurement, green circle for nasal washes and rectal swab sample collection, and blue circle indicates nasal turbinate and lung tissue harvest for viral titration. **b** Timetable for ferret-to-ferret transmission study and blood sample collection for serum neutralizing and IgG antibody titration: at 24 h post-infection of the infected ferret groups, 12–24 month-old naive ferrets (*n* = 3/group) were introduced in direct contact with infected ferrets and was co-housed with each group of infected ferrets in the same cage up to 12 days. Several time points are indicated for monitoring and sample collection. Orange circle denotes time point for temperature and body weight measurement, while purple circle indicates points for collection of nasal washes for viral titration. Red water drop shape indicates euthanasia after whole blood collection. **c** Timetable for RNA-seq analysis: groups of ferrets (*n* = 6/group) were SARS-CoV-2 inoculated at 0 dpi with exception to the mock infection group. PBS-treated ferrets (*n* = 9) were intranasally inoculated with phosphate-buffered saline (PBS) for the mock-infection group. Ferrets of the same ages as the test groups (*n* = 9 (Juvenile = 3, young adult = 3, aged = 3)) were then euthanized at 2 dpi, and lungs were harvested for RNA-seq analysis. SARS-CoV-2 infected ferrets by age (*n* = 3/group) were euthanized at 2 dpi and 5 dpi respectively, and lungs were harvested for RNA-seq analysis.

**Fig. 2:**
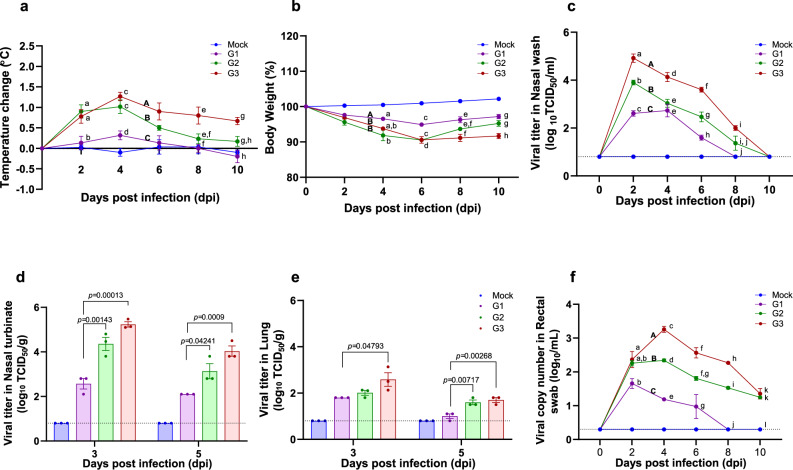
Pathogenicity of SARS-CoV-2 infection among different ages of ferrets. Groups of ferrets [under 6 months (G1), one to two years old (G2), and more than three years old (G3); *n* = 9 per group] were inoculated with 10^5.8^ TCID_50_ of NMC-nCoV02 strain by the intranasal route. All groups were observed for morbidity and mortality for 10 days. The temperature change (**a**) and weight loss (**b**) were monitored. To compare virus growth in respiratory tracts, nasal washes (**c**) were collected at 0, 2, 4, 6, 8, and 10 dpi. Nasal turbinate (**d**) and lung (**e**) tissues were collected to recover infectious virus from infected ferrets (*n* = 3) at 3 and 5 dpi. To compare virus growth in gastrointestinal tracts, rectal swabs (**f**) were collected at 0, 2, 4, 6, 8, and 10 dpi. Infectious viral titers in nasal washes and tissue specimens (**c**, **d** and **e**) were measured in Vero Cells, and viral RNA copy numbers in rectal swabs were quantitated using real-time PCR (**f**). Data are presented as mean values ± SEM. Groups with the same letter are of the same subgroup in the post-hoc analysis. Lower case letters indicate significant differences at each time point (*P* values are a vs b: 0.00398 or 0.02009, c vs d: 0.00335 or 0.00011, e vs f: 0.038 and g vs h: 0.0033 (a), a vs b: 0.00476, c vs d: 0.0045 or 0.0047, e vs f: 0.00807 and g vs h: 0.00207 or 0.02917 (b), a vs b: <0.0001, a vs c: <0.0001, b vs c: <0.0001, d vs e: 0.00017 or 0.00247, f vs g: 0.0011, f vs h: <0.0001, g vs h: 0.0067 and i vs j: 0.0038 (c), a vs b: 0.015, c vs d: <0.0001, c vs e: <0.0001, d vs e: <0.0001, f vs g: 0.00266, h vs i: <0.0001, h vs j: <0.0001, i vs j: <0.0001 and k vs l: 0.00016 or <0.0001 (f)), while upper case letters indicate significant differences of area under the curve as change over the entire period (*P* values are A vs B: < 0.0001, A vs C: < 0.0001 and B vs C: < 0.0001 (**a**), A vs B: < 0.0001 (**b**), A vs B: < 0.0001, A vs C: < 0.0001 and B vs C: < 0.0001 (**c**), A vs B: < 0.0001, A vs C: < 0.0001 and B vs C: < 0.0001 (**f**)). The area under the curve was calculated using Kruskal–Wallis with Bonferroni as a post-hoc analysis. Source data are provided as a Source Data file.

To elucidate the clinical manifestations of SARS-CoV-2 infection across different age groups, ferrets (*n* = 9/group) were inoculated with 10^5.8^ of 50% tissue culture infective dose (TCID_50_)/mL of NMC-nCoV02 strain through the intranasal (IN) route (Fig. 1a). Body weight and temperature were measured before infection and every day after infection until 10 days post-infection. While ferrets less than six months of age (G1) showed no increase in temperature, both 1–2 year-old (G2) and more than three-year-old (G3) groups of SARS-CoV-2 infected ferrets showed elevated temperatures at 2–6 dpi, where the G3 group showed a prolonged elevated temperature even at 10 dpi (Fig. 2a). This trend was also associated with changes in body weight, where the G1 group showed less than 5% weight loss during the entire SARS-CoV-2 infection period, while the G2 and G3 groups showed a maximal 10% weight loss at 6 dpi, followed by a rapid recovery of the G2 group from 6 dpi but not the G3 group (Fig. 2b). To compare clinical manifestations of SARS-CoV-2 infection, we developed a clinical assessment scoring system to evaluate clinical symptom (CS) values based on a 20-min observation period of cough, rhinorrhea, and reduced activity. These CS values were compared among ferret groups as described in Table 1 and Supplementary Table 1. The G2 and G3 groups showed the highest CS values of 4.17 and 4.67 at 4 dpi, respectively, while the G1 group showed a maximal CS value of 1 at 2 dpi before quickly recovering within four days, exhibiting a mild to asymptomatic infection (Table 1 and Supplementary Table 1). In particular, the G3 group showed a prolonged period of high CS value that lasted until 10 dpi. Collectively, these results revealed that aged ferrets exhibit more severe and persistent clinical features of SARS-CoV-2 infection compared to younger ferrets.

**Table 1:** Clinical scores of ferrets infected with SARS-CoV-2.

Group		0 dpi	2 dpi	4 dpi	6 dpi	8 dpi	10 dpi
G1 (Juvenile)	Cough	0.00	0.00	0.00	0.00	0.00	0.00
	Runny nose	0.00	0.56 ± 0.50	0.33 ± 0.47	0.00	0.00	0.00
	Movement, activity	0.00	0.44 ± 0.50	0.17 ± 0.37	0.00	0.00	0.00
	Total	0.00	1.00	0.50	0.00	0.00	0.00
G2 (Young adult)	Cough	0.00	0.33 ± 0.47	1.00	1.00	0.00	0.00
	Runny nose	0.00	0.22 ± 0.42	1.33 ± 0.47*	1.00	0.00	0.00
	Movement, activity	0.00	0.67 ± 0.67	1.83 ± 0.37**	1.67 ± 0.47***	1.00	0.00
	Total	0.00	1.22	4.17	4.00	1.00	0.00
G3 (Aged adult)	Cough	0.00	0.56 ± 0.50^†^	1.00	1.00	0.00	0.00
	Runny nose	0.00	0.44 ± 0.68	1.67 ± 0.47^††^	1.33 ± 0.47^†††^	0.67 ± 0.47	0.33 ± 0.47
	Movement, activity	0.00	0.78 ± 0.92	2.00**	2.00	1.00	0.67 ± 0.47
	Total	0.00	1.78	4.67	4.00	1.67	1.00

Observational clinical symptoms: Cough, rhinorrhea, movement, and activity. Score: 0; normal, 1: occasional, mild reduced activity, 2: frequent, reduced activity. Scores were measured by observation of clinical symptoms for at least 20 min in each group of ferrets based on the following criteria: Cough: 0; no evidence of cough, 1; occasional cough, 2; frequent cough (score 2). Rhinorrhea: 0; no nasal rattling or sneezing, 1; moderate nasal discharge on external nares, 2; severe nasal discharge on external nares. Movement, activity: 0; normal movement and activity, 1; mild reduced movement and activity, 2; evidence of reduced movement and activity.

Asterisks and daggers indicate statistical significance compared with G1 (Juvenile) as evaluated by two tailed *t*-tests and nonparametric tests (*p = 0.0073, **p < 0.0001, ***p = 0.0075, ^†^*p* = 0.006, ^††^*p* = 0.0012 and ^†††^*p* = 0.0161).

### Comparison of viral titer and shedding period among ferrets of different ages

To evaluate whether the clinical features seen in the ferret groups were associated with the degree of virus replication, nasal washes were collected every day for 10 days to measure the infectious viral titers (Fig. 2c). SARS-CoV-2 was isolated from all infected ferrets, regardless of their ages, from 2 to 6 dpi, whereas ferrets in the G2 and G3 groups continued to shed infectious virus until 8 dpi. Comparison analysis of the viral titers revealed that the G3 group showed higher viral titers (3.5 to 4.9 log_10_TCID_50_/mL) from 2 to 6 dpi than the G1 and G2 groups. While the G2 group showed higher viral titers at 2 dpi than the G1 group, comparable viral titers were observed thereafter. To further examine the viral titer in respiratory organs of infected animals, three ferrets from each group were euthanized at 3 and 5 dpi for measurement of viral titers in the nasal turbinate and lungs. As expected, the G3 group showed the highest viral titer among the groups with a maximum of 5.2 log_10_TCID_50_/g in nasal turbinate at 3 dpi. The G2 group also showed higher viral titers compared to the G1 group (Fig. 2d). Consistently, the G3 group showed a higher viral titer (2.6 log_10_TCID_50_/g) in lung specimens compared to the G1 and G2 groups at 3 dpi (Fig. 2e). These results clearly demonstrate that aged ferrets (G3) shed significantly higher amounts of infectious virus in their nasal discharge for a longer period of time than younger ferrets, correlating with the severity of clinical disease.

As gastrointestinal involvement has also been documented during coronavirus infection of animals and humans^35,36^, we collected fecal specimens from the infected ferrets and performed qRT-PCR to assess SARS-CoV-2 viral loads and shedding periods from the intestines of ferrets of different ages (Fig. 2f). While viral RNAs were detected in fecal specimens of all three groups from 2 to 4 dpi, the G3 group showed the highest viral RNA copy number from 2 to 8 dpi, followed by the G2 group. On the other hand, the G1 group showed a small peak of viral RNA at 2 dpi, which rapidly declined thereafter to an undetectable range at 6 dpi. These results demonstrate that aged ferrets show significantly higher levels of SARS-CoV-2 RNA in the gastrointestinal tract compared to juvenile and young adult ferrets.

### Differential lung histopathology of infected ferrets of different ages

**Fig. 3:**
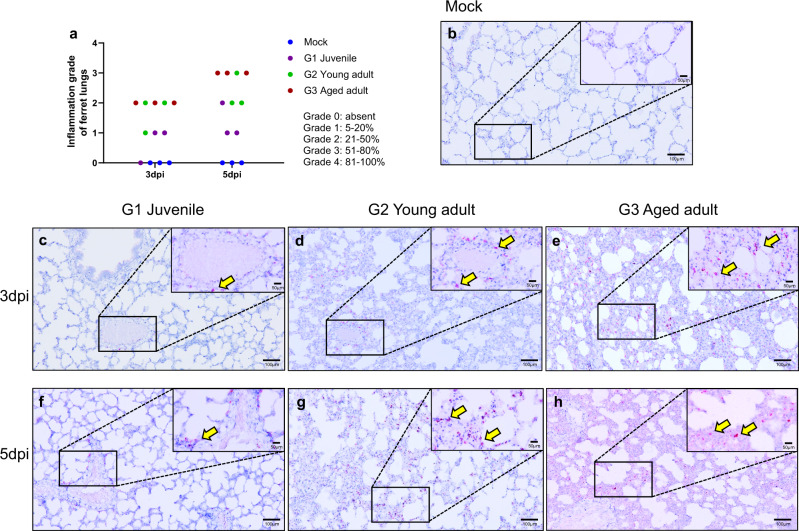
RNAscope in situ hybridization in the lung of SARS-CoV-2 infected ferrets. To detect the SARS-CoV-2 RNA (Spike gene) in lung tissues, RNAscope in situ hybridization was performed using a Spike-specific probe and visualized using RNAscope 2.5 HD Reagent Kit RED. The degree of inflammation in lung tissues was scored as Grade 0–4 based on the estimated percentage of the lung tissue slides affected by lesions (*n* = 6/group) (**a**). SARS-CoV-2 spike RNA-positive cells (Yellow arrows) in lung tissues of mock infected (**b**), juvenile ferrets (≤6 months, G1 group) (**c** and **f**), young adult (1 ≤ age ≤2 years, G2 group) (**d** and **g**), and aged ferrets (3-year ≤ ages) (**e** and **h**). Lung sections of different age groups at 3 dpi (*n* = 3/group) (**c**, **d**, and **e**) and at 5 dpi (*n* = 3/group) (**f**, **g**, and **h**). Magnification is 100x and scale bar represents 100 μm. Insert indicates the magnified (200x) of SARS-CoV-2-positive and scale bar represents 50 μm.

COVID-19 has most commonly been shown to be associated with a wide range of pulmonary manifestations^37^. The aged ferrets showed considerable inflammation affecting most parts of lungs compared to the juvenile and young adult ferrets that showed only mild to moderate inflammation. Notably, all aged ferrets showed more than 50% lung damage at 5 dpi, (Fig. 3a and Supplementary Fig. 3). To further assess the extent of pulmonary damage among ferrets of different ages, RNAscope in situ hybridization and histopathological examination were conducted in the lung specimens (Fig. 3 and Supplementary Fig. 2). RNAscope analysis detected more SARS-CoV-2 infected cells in young adult and aged ferrets compared to the juvenile ferrets (Fig. 3c–h). Specifically, both young adult and aged ferrets showed significantly higher numbers of viral RNA-positive cells compared to juvenile ferrets at 3 and 5 dpi (Supplementary Fig. 2a). However, there was no significant difference between young adult and aged ferrets at 5 dpi (Supplementary Fig. 2), which was reflected by the virus titers in the lungs (Supplementary Fig. 2e). The G2 group (Supplementary Fig. 3e–h) displayed only moderate pathological changes in the lung in comparison to the G1 group (Supplementary Fig. 3a–d). In contrast, analysis of lungs from the G3 group revealed increased severity of pathological features highlighted by the increased inflammatory cell infiltration and alveolar septa being widened, edematous, and congested (Supplementary Fig. 3i–l). To rule out differential ACE2 expression among age groups, which may affect disease manifestation and virus replication kinetics in ferrets, ACE2 RNAscope analysis was performed on the lung sections, which did not show any significant difference of ACE2 expression among different age groups (Supplementary Fig. 4). These results demonstrate that the severity of lung damage is closely associated with the number of SARS-CoV-2 RNA-positive cells in the lungs, but not with ACE2 receptor expression levels.

### Differential SARS-CoV-2 neutralization antibody titers and serum IgG among ferrets of different ages

**Fig. 4:**
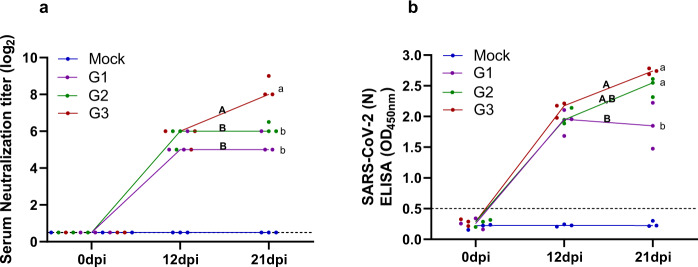
Serum neutralizing antibody (NAb) and IgG antibody titers in different ages of ferrets. The NAb titers against SARS-CoV-2 NMC-nCoV02 (100 TCID_50_) among different age groups was measured in Vero cells with serially diluted ferret sera collected at 0, 12, and 21 dpi (**a**). The IgG titers among different age groups in serially diluted sera collected at 0, 12, and 21 dpi (**b**). Groups with the same letter are of the same subgroup in the post-hoc analysis. Lower case letters indicate significant differences at each time point (*P* values are a vs b: 0.00017 or 0.00168 (**a**), a vs b: 0.03 or 0.0044 (**b**)), while upper case letters indicate significant differences of area under the curve as change over the entire period (*P* values are A vs B: 0.0012 or 0.0233 (**a**), A vs B: 0.03 (**b**)). The area under the curve was calculated using Kruskal–Wallis with Bonferroni as a post-hoc analysis. Data were analyzed in GraphPad Prism 9.1.2. Data are presented as mean ± SEM (*n* = 3). The limit of detection for NAb titers and OD_450 nm_ value is 0.5, indicated by the dotted line. Source data are provided as a Source Data file.

To compare the serum neutralization antibody (NAb) titers among ferrets of different ages, blood was collected from each group of ferrets at 12 and 21 dpi (Fig. 1b). At 12 dpi, all tested ferrets showed NAb titers of 32–64 without significant differences between the groups. However, at 21 days, the NAb titers were at least four-fold higher in the G3 group (GMT 256) than those in the G1 group (GMT 64) (Fig. 4a). Furthermore, the G3 group showed increased NAb titers at 21 dpi compared to 12 dpi, suggesting continuous activation of the immune response as long as 21 dpi (Fig. 4a). We further examined the serum IgG antibody titer from each group. The results revealed that the young adult (G2) and aged adult (G3) groups showed increased anti-SARS-CoV-2 antibody titers at 21 dpi (Fig. 4b). However, there were no statistical differences of anti-SARS-CoV-2 antibody titers between the juvenile group at 12 and 21 dpi. Two ferrets of the juvenile group (G1, *n* = 3) exhibited a decrease of anti-SARS-CoV-2 antibody titers, and only one ferret showed a similar anti-SARS-CoV-2 antibody titer at 12 dpi. This indicates that similar to severe COVID-19 patients^38^, aged ferrets display severe clinical symptoms and high viral titers in the respiratory tract as well as high serum NAb and IgG responses.

### Comparison of SARS-CoV-2 transmission in ferrets by age

As viral titers differed significantly among different age groups, naïve ferrets (*n* = 3/group, 12–24 month-old) were co-housed and placed in direct contact (DC) with infected ferrets 24 h after the primary infection to compare the transmission efficiency of SARS-CoV-2 (Fig. 1b). In parallel with the infected ferrets, clinical manifestations of SARS-CoV-2 infection were also monitored in DC ferrets until 11 days post-contact (dpc). The DC ferrets co-housed with aged (G3 group) animals showed the highest and most prolonged CS value compared to DC ferrets co-housed with those in the young adult (G2 group) (Supplementary Table 2). However, DC ferrets co-housed with the juvenile animals (G1 group) did not show any symptoms of clinical disease during the course of infection (Supplementary Table 2). While a slight elevation in body temperature was observed among contact ferrets co-housed with aged and young adult animals, no significant increase in body temperatures was noted in contact ferrets co-housed with juvenile animals (Supplementary Fig. 1a). Of note, there was no loss of body weight in any of the contact groups (Supplementary Fig. 1b).

**Fig. 5:**
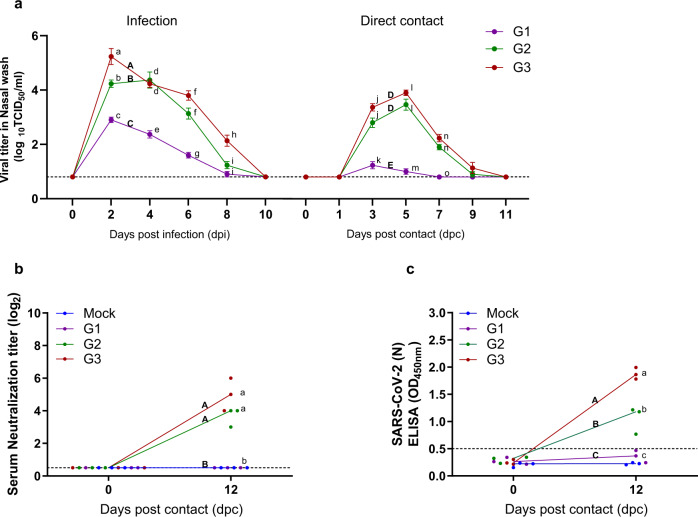
Transmission properties of SARS-CoV-2 in ferrets of different ages upon co-housing naïve ferrets. Naïve ferrets (*n* = 3/group) were exposed to direct contact (DC) with each group of infected ferrets of different ages (*n* = 3/group) starting 24 h after the primary infection, followed by measurement of their viral titers from nasal washes on day 1, 3, 5, 7, 9, and 11 post contact (**a**). Serum neutralizing antibody titers in contact groups at 12 dpc (**b**). IgG titers in contact groups at 12 dpc (**c**). Data are presented as mean values ± SEM. Groups with the same letter are of the same subgroup in the post-hoc analysis. Lower case letters indicate significant differences at each time point (*P* values are a vs b: 0.00897, a vs c: 0.00046, b vs c: 0.03398, d vs e: 0.0013 or 0.0019, f vs g: 0.00175 or 0.00024, h vs i: 0.0036 or 0.0171, j vs k: 0.00088 or 0.00015, l vs m: <0.0001 and n vs o- 0.00058 or 0.00013 (**a**), a vs b: 0.0009 or <0.0001 (**b**), a vs b: 0.0031, a vs c: <0.0001 and b vs c: 0.0053 (**c**)), while upper case letters indicate significant differences of area under the curve as change over the entire period (*P* values are A vs B: 0.00844, A vs C: < 0.0001, B vs C: 0.00014 and D vs E: 0.0001 or <0.0001 (**a**), A vs B: 0.0009 or <0.0001 (**b**), A vs B: 0.0031, A vs C: < 0.0001 and B vs C: 0.0053 (**c**)). The area under the curve was calculated using Kruskal–Wallis with Bonferroni as a post-hoc analysis. Data were analyzed in GraphPad Prism 9.1.2. Source data are provided as a Source Data file.

Furthermore, infectious virus titers were measured in the nasal washes (Fig. 5a). Virus was detectable in nasal washes of the DC ferrets as early as 3 dpc, where the DC ferrets exposed to the G3 group showed the highest virus titers at all time points with a peak of 4.10 log_10_ TCID_50_/mL at 5 dpc. The DC ferrets co-housed with G2 and G3 groups shed infectious virus up to day 9 post-contact, whereas the DC ferrets exposed to G1 group showed the lowest virus titers throughout the co-housing periods, reaching an undetectable range of infectious virus after 7 dpc (Fig. 5a). Overall, these results demonstrate that as aged ferrets harbored high SARS-CoV-2 titers in their respiratory tracts, they were able to effectively transmit the virus to naïve ferrets by direct contact. Consistent with children who appeared to be less infectious than adults infected with SARS-CoV-2 due to their mild clinical manifestations of disease^39^, the infected juvenile ferrets carried low virus titers and was not readily infectious to naïve ferrets upon direct contact.

To compare the immune responses elicited by the direct contact ferret groups, ELISA and serum neutralization assay were performed to measure the IgG and serum neutralization antibody (NAb) titers, respectively (Fig. 1b). While NAb was below the detection limit (below 0.5 log_2_) in G1-exposed DC ferrets at 12 dpi (Fig. 5b), G2- or G3-exposed DC ferrets exhibited moderate and significant increases in NAb titers, respectively. In addition, IgG antibody titers showed a similar pattern to the NAb response. The G1-exposed ferrets had IgG titers below the level of detection (less than 0.5 optical density 450 _nm_ value), whereas G2 or G3-exposed ferrets showed elevated IgG titers (Fig. 5c). Together, in comparison with ferrets contacted with young and juvenile groups, ferrets contacted with aged groups exhibited prolonged clinical manifestations of infection, shedding higher viral titers through the respiratory tract, and mounting higher NAb titers, and increased IgG antibody immune responses.

### Transcriptional profile of immune-related genes in lung tissues of SARS-CoV-2-infected ferrets

**Fig. 6:**
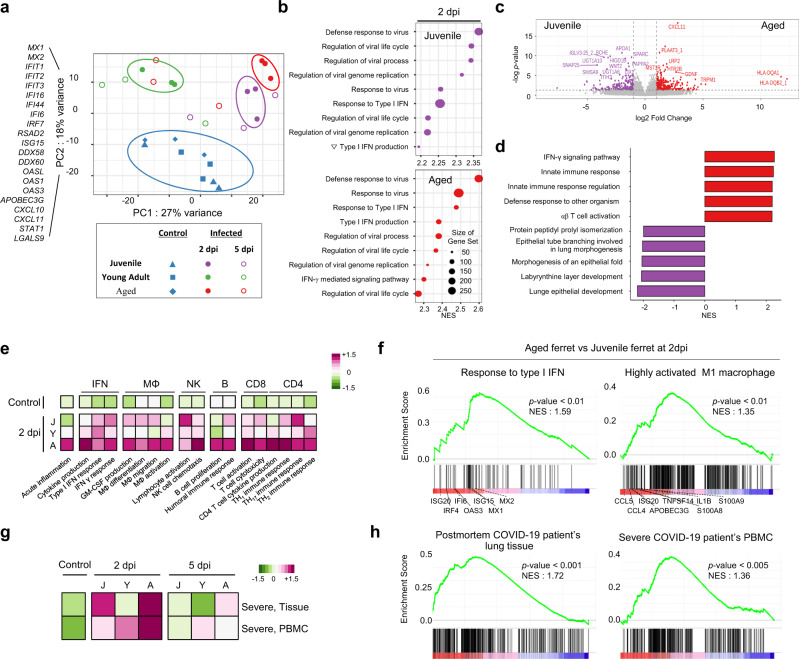
Transcriptional profile of immune-related genes in the lung of SARS-CoV-2-infected ferrets. **a** The overall variation of transcriptional profiles in the lung of SARS-CoV-2-infected (*n* = 3/group) and age-matched uninfected (total *n* = 9) ferrets using principal component analysis (PCA). **b** The ‘Common’ gene sets were composed of genes differentially upregulated in more than two groups, while ‘Juvenile-specific’, ‘Young adult-specific’ and ‘Aged-specific’ gene sets were composed of genes uniquely upregulated in each group. Representative immune-related genes were listed next to the heatmap. Plots with normalized enrichment score (NES) from enrichment analysis of representative Gene Ontology (GO) biological pathway in juvenile and aged ferrets at 2 dpi, compared to age-matched control ferrets. **c** Volcano plots showing DEGs between juvenile and aged ferrets at 2 dpi. **d** Bar plots with NES from enrichment analysis of GO biological pathway in juvenile and aged ferrets at 2 dpi. **e** Heatmap of gene set variation analysis (GSVA) with immune-related GO biological pathway. J Juvenile, Y Young adult, A Aged. **f** Gene set enrichment analysis (GSEA) of gene sets related to type I IFN response and highly activated M1 macrophage between juvenile and aged ferrets at 2 dpi. The Y axis represents the enrichment score (ES), which reflects the degree to which a gene set is overrepresented at the top or bottom of a ranked list of genes, and the individual bar represents where the individual gene of each gene set is located in the ranked list of genes, corresponding to the projection of the gene set on the red-to-blue gradient, with red representing higher expression and blue representing lower expression (**f** and **h**). The *p* value of GSEA is the probability under the null distribution calculated by the one-sided permutation test (f: *p* = 0.0062, 0.0051 and h: *p* < 0.0001, 0.0025). GSVA and GSEA with indicated gene sets from severe COVID-19 patients between young adult and aged ferrets at 2 dpi. Control is the mean value (*n* = 9) of juvenile, young adult, and aged control ferrets. Heatmap colors from green to magenta represent low to high enrichment, respectively. While changes in gene expression are conveyed using color gradation (green, light green, pink, and magenta), and unchanged expression is represented by white (**e** and **g**). Source data are provided as a Source Data file.

To gain a comprehensive understanding of the transcriptional profile among ferrets of different ages following SARS-CoV-2 infection, we performed RNA sequencing analysis of lung tissues from the G1, G2, and G3 ferret groups at 2 and 5 dpi, and compared the results with those of non-infected age-matched ferrets (control, *n* = 9 (juvenile = 3, young adult = 3, and aged = 3)) (Fig. 1c). We first analyzed the overall variation of the samples using principal component analysis (PCA). Distinct clusters were observed among G1, G2, and G3 groups at 2 dpi (filled circle, ), which were also clearly separated from that of control ferrets (filled blue symbols, ) (Fig. 6a). Although, no age-dependent differences in gene clustering were observed among the nine control ferrets, intriguingly, SARS-CoV-2 infected ferrets and control ferrets were differentially clustered by age (Fig. 6a). This effect was attributed to principal component (PC) 2, largely composed of interferon-stimulated genes (ISGs), such as *MX1*, *IRF7*, *ISG15*, and *OAS1* (Fig. 6a, Supplementary Table 3). Further, age-dependent differences, especially between young adult and aged infection groups animals, were observed in PC 1, which is composed of regulation of cilium movement-related genes such as *DNAH11*, *DNAAF1*, *CFAP43*, and *CCDC40*. These findings indicate that genes related to IFN responses are highly enriched in infected ferrets during the early stage of SARS-CoV-2 infection. Compared with 2 dpi, the samples at 5 dpi (open circle) did not display any particular clustering by age in PCA.

To gain a deeper insight into the divergent immune responses of SARS-CoV-2-infected ferrets with age, we identified differentially expressed genes (DEGs) in each group (Supplementary Fig. 5a, Supplementary Table 4). Compared to age-matched PBS-treated control ferrets, a total of 61 genes, including a number of ISGs (*IFI44L, ISG15, ISG20, IFIT1, IFIT2, MX1, MX2, OAS1, OAS3*, and *OASL*), chemokines (*CXCL11*), and interferon regulatory transcription factor (*IRF7*), were commonly upregulated in SARS-CoV-2 infected-ferrets at 2 dpi, regardless of age (Supplementary Fig. 5a). On the other hand, 142, 67, and 176 genes were uniquely upregulated in G1, G2, and G3 groups, respectively, at 2 dpi (Supplementary Fig. 5a). The DEGs that were specifically upregulated in the G3 group at 2 dpi included genes related to inflammatory response (*C4A*, *BATF2*, *CCL3*, and *CXCL10*). In addition, several genes related to immune activation and inflammation (*TNFSF18, CCL8, and IL-17F*) were specifically upregulated in the G2 group at 2 dpi (Supplementary Fig. 5a). At 5 dpi, various ISGs (*IFI6, IFIT1, OAS1, OAS3*, and *OAS*L) and innate immunity-associated genes (*CLEC4G*, *RSAD2*, and *TRIM22*) were commonly upregulated in all SARS-CoV-2 infected ferrets (Supplementary Fig. 5a). Gene Set Enrichment Analysis (GSEA) revealed that DEGs of the infected ferrets were highly enriched with gene sets related to the anti-viral innate immune response at 2 dpi (Fig. 6b and Supplementary Fig. 5b). These immune activation features were maintained even at 5 dpi, especially in G3 (Supplementary Fig. 5c). In contrast, tissue remodeling-related gene sets, such as intraciliary transport, microtubule organizing center organization, and cilium movement, were highly enriched in the G1 juvenile group at 5 dpi (Supplementary Fig. 5c), which may be closely associated with matrix remodeling during the recovery of lung epithelial injury in the juvenile group.

Notably, direct comparison of the DEGs between G1 and G3 groups at 2 dpi revealed upregulated genes in the G3 group were mainly composed of genes related to active inflammation, such as CXCL11 and CXCL10 (Fig. 6c). Even though CXCL11 was upregulated in all infected ferrets compared to the control group, the aged ferrets exhibited markedly more CXCL11 upregulation compared to the juvenile group. In contrast, upregulated genes in the G1 group included genes related to tissue remodeling and tissue macrophages, such as *APOA1*, *SPARC,* and *COL12A1* (Fig. 6c). GSEA revealed that DEGs the G3 group were highly enriched with gene sets related to both innate and adaptive immune responses compared to those of the G1 group (Fig. 6d).

Furthermore, Gene Set Variation Analysis (GSVA) with public gene sets revealed that genes related to B cell response and T cell response were predominantly enriched in the aged group (G3) at 2 dpi, compared to the juvenile (G1) or the young adult (G2) ferrets (Fig. 6e), which is in agreement with a recent clinical study that showed stronger antibody and T cell responses in severe COVID-19 patients than in patients with mild disease^40,41^. Moreover, other immune-related gene sets, including IFN response, and macrophage and NK cell activation, were also highly enriched in the aged group at 2 dpi (Fig. 6e). Notably, gene sets related to type I IFN responses and activated M1 macrophages were significantly enriched in aged ferrets at the earlier stage of SARS-CoV-2 infection (Fig. 6f), suggesting a marked activation of innate immune response promptly ensued from the initial infection in aged ferrets. Moreover, aged ferrets showed high expression of chemokines (CCL4 and CXCL10) and inflammatory cytokines (IFNB1, IL1B, IL6, and IL7) at 2 dpi compared to other groups (Supplementary Fig. 6). These data suggest that aged ferrets have increased expression of inflammatory cytokines and chemokines during the early phase of infection. Finally, we investigated whether the SARS-CoV-2-infected aged ferrets also reproduced the natural course of infection and severity of COVID-19 as seen in humans. Clearly, the gene sets upregulated in postmortem lung tissue of COVID-19 patients^42^ or PBMCs from severe COVID-19 patients^43^ were highly enriched in aged ferrets (G3), especially at 2 dpi, compared to the juvenile (G1) and young adult (G2) ferrets (Fig. 6g, h). In addition, when we analyzed the intersective and uniquely upregulated DEGs between 2 and 5 dpi in each age group, we identified that gene sets related to anti-viral responses, such as “defense response to virus”, were commonly enriched within the “Intersective gene set” and “2 dpi only” in all age groups, but not in the “5 dpi only”. Furthermore, upregulated DEGs in aged ferrets at 2 dpi were highly enriched for gene sets related to inflammatory and anti-viral responses, which differed from that of juvenile and young adult ferrets (Supplementary Fig. 7). These transcriptional profiles of immune-related genes indicate that the SARS-CoV-2-infected ferret model reflects the immunological properties and age-dependent severity of COVID-19 in humans.

## Discussion

SARS-CoV-2 has already had devastating effects on the global community affecting myriad aspects of our lives. While effective vaccines have been developed, the exact timeline that it could reach the entire general public to put an end to the pandemic is still unclear. Although there is finally hope on the horizon, things are expected to get worse in the meantime, as thousands of people die every day and are separated from their families, pushing the national health care system to the brink. Furthermore, emergence of new variants, such as the Brazil variant (P.1), UK variant (B.1.1.7), South African variant (B.1.351), and recently the California variant (B.1.427/B.1.429), increases public health concern whether they could abrogate the effectivity of the existing SARS-CoV-2 vaccines. Moreover, as part of the current social distancing policy, student attendance in academic institutions is restricted in many countries and is being replaced with online classes. However, recent studies have suggested that although children and young adult populations may predominantly exhibit asymptomatic SARS-CoV-2 infections with low pathogenicity, they may still be carriers of infectious viruses^44,45^. In contrast, majority of patients with severe morbidity and mortality are reportedly elderly people who have limited social activities compared to other age groups^46–48^. It became evident that the wide range of disease severity and the sequelae of SARS-CoV-2 infection are correlated with both the age of the afflicted person and presence of pre-existing medical conditions. Thus, to investigate the differential and diverse clinical manifestations in COVID-19 patients of different ages, age-related disease severity of COVID-19 was assessed in ferrets of three different age groups. Previous studies reported that SARS-CoV-2 infected ferrets are asymptomatic or exhibit mild clinical signs with lung pathology and viral shedding patterns similar to asymptomatic and mild human cases of COVID-19^31,38^. On the other hand, Kim et al. reported clear clinical symptoms, such as body weight loss and increased temperature, in SARS-CoV-2 infected ferrets^28^. Although there are several differences among these studies, including virus strains and infection dose, one of the main differences is animal age. Thus, the age of asymptomatic ferrets was less than 6 months^38^, while symptomatic SARS-CoV-2 was seen in relatively older ferrets, 12–20-month-old animals^28^, compared with the previous two studies^28,38^. In the current study, we confirm that the observed differences in clinical symptoms are closely associated with animal age. Although there is currently no apodictic formula to calculate age equivalency between ferrets and humans, various reports indicate that average life span of domesticated ferrets is between 5 and 7 years. However, given their short life span and the observed onset of serious health problems as early as 3–4 years of age in most ferrets, veterinarians considered ferrets to be geriatric at 3 years of age^49^. Thus, although we cannot accurately correlate the lifespan and developmental stages of ferrets and humans, we believed that ferrets more than 3 years old could represent the aged human population. Although a recent study reported that males are more susceptible to SARS-CoV-2 infection^50,51^, in the current study we did not identify any sex-related differences in SARS-CoV-2 pathogenesis in the ferret animal model, since we only used female ferrets. However, further study will be needed to elucidate any sex-dependent effects on pathogenesis and immune responses between age groups.

In this study, we demonstrate the age-associated pathogenesis of SARS-CoV-2 infection using a ferret model. Comparison analysis of clinical symptom values and viral loads showed that the aged ferret model fully recapitulates clinical manifestations of COVID-19 in humans (Table 1 and Fig. 2). Recent reports of human patients with SARS-CoV-2 infection in China indicate that aged and comorbid patients carried higher viral loads and experienced difficulty recovering from severe pneumonia, which eventually led to higher mortality^52^. These are in line with findings from our aged ferret model. In particular, aged ferrets showed higher lung damage scores, increased viral loads in their respiratory tracts, and higher virus shedding compared to the juvenile and young adult ferrets. Moreover, RNAscope in situ hybridization assay clearly demonstrated that aged ferret group contained higher numbers of SARS-CoV-2 RNA-positive lung cells and infiltrating inflammatory cells compared to juvenile and young adult groups, which is correlated with the severity of viral pathogenesis in the aged ferret group. We found no detectable difference of the ACE2 expression among different age groups, ruling out the possibility that differential ACE2 expression among different age groups might affect disease manifestation and virus replication kinetics in ferrets.

In addition, aged ferrets showed high expression of chemokines (CCL4 and CXCL10) and inflammatory cytokines (IFNB1, IL1B, IL6, and IL7) in the early phase of infection. These mediators may contribute to the aberrant inflammatory response, which in turn causes severe pulmonary pathologies in aged adult ferrets. These findings partially correlate with recent reports of severe COVID-19 cases with increased expression of pro-inflammatory cytokines and chemokines, which are associated with pulmonary inflammation and extensive lung damage^53,54^. Cytokine storm is potentially a life-threatening event during the course of COVID-19. Patients with severe COVID-19 often exhibit acute respiratory distress syndrome, a consequence of cytokine storm resulting from the marked expression of a combination of immune-active molecules. In this study, the pathology of SARS-CoV-2 in infected aged ferrets considerably reflects that of aged COVID-19 patients, making it a valuable animal model to understand the age-dependent viral pathogenesis of SARS-CoV-2. Importantly, the ferrets directly infected with SARS-CoV-2 showed the highest viral titer at 2 dpi, while ferrets infected by direct contact transmission showed moderate virus titers at 3 dpc followed by peak titers at 5 dpc in all groups, with the exception of the juvenile ferret-exposed group. This differential infection and transmission phenomenon between groups may be explained by the initial infection dose they are exposed to. The direct infection groups were infected with a high dose of virus (10^5.8^ TCID_50_/mL, which is almost the maximum titer in ferret respiratory tracts) and showed a gradual decrease of virus titer after the maximum titer was reached at 2 dpi. In contrast, ferrets infected by direct contact were presumably infected with lower titers, resulting in naïve animals reaching maximum virus titer on 5 dpc. Therefore, the natural infection pattern of SARS-CoV-2 may be more similar to that of the direct contact groups. For example, the juvenile group was presumably exposed to lower amounts of infectious virus which were rapidly cleared in naïve animals.

The role of juveniles (<18 years of age) in the spread of SARS-CoV-2 has not been fully defined. A recent study shows that children are not likely to be the source of SARS-CoV-2 transmission and outbreak, and thus, are minor drivers of the COVID-19 pandemic^36^. In contrast, juveniles typically exhibit the highest rates of influenza virus infection and are considered to play a critical role in influenza virus spread. It is believed that because juveniles exhibit mild clinical manifestations of COVID-19, they are less likely to transmit infectious SARS-CoV-2 than adults who exhibit more severe symptoms. In support of these reports, our present study shows that the infected juvenile ferrets carry low virus titers and are not readily infectious to naïve contact animals. This indicates that besides aged ferrets, juvenile ferrets can also potentially be a useful animal model in understanding the important scientific aspects of the infrequent transmission of SARS-CoV-2 from children to other children and from children to adults.

Although the kinetics of antibody development in SARS-CoV-2 infected ferrets were comparable among all three groups at 12 dpi, NAb titers were two- and four-fold higher in the young adult and aged ferret groups, respectively, than in the juvenile group (Fig. 4). It is noteworthy that the aged ferret group, which manifested more severe clinical symptoms along with the highest viral loads in the respiratory tract, showed a four-fold increase in NAb titer at 21 dpi over that at 12 dpi, suggesting a close association between clinical outcomes and antibody production. This result is also well in agreement with a recent study^54^ reporting that SARS-CoV-2 serum neutralizing antibody levels were higher in severe SARS-CoV-2 patients than in asymptomatic or mild patients. Thus, SARS-CoV-2-infected aged ferrets can also be used to understand the immunological aspects of the high neutralizing antibody titer in patients with more severe COVID-19.

Recently, a transcriptome analysis study of COVID-19 patients demonstrated robust induction of type I IFN responses in severe COVID-19 patients compared to mild/moderate COVID-19 patients^42^. In this study, transcriptome analysis during the early stage of SARS-CoV-2 infection in ferrets also revealed enrichment of genes involved in both innate and adaptive immune responses in aged ferrets compared to juvenile and young adult ferrets. Notably, the gene sets related to the type I IFN response and activated M1 macrophages were significantly enriched in SARS-CoV-2-infected aged ferrets at 2 dpi. Furthermore, infected aged ferrets showed enrichment of genes that were also expressed in tissues of patients with severe COVID-19, such as lung tissues of post-mortem COVID-19 patients and PBMCs from patients with severe COVID-19 symptoms^42,43^. While the anti-viral response was commonly seen from 2–5 dpi, it was most apparent at 2 dpi. Also, the occurrence of stronger inflammatory and prolonged immune responses in aged ferrets at 2 dpi may affect pathogenic features of SARS-CoV-2 infection (Supplementary Fig. 7). These results indicate that the SARS-CoV-2-infected aged ferrets not only recapitulate the prevalence and disease course of severe symptoms seen in COVID-19 patients, but also the corresponding alterations of transcriptional landscape of COVID-19 among different age groups. In particular, the aged ferrets demonstrated upregulation of genes related to early activation of an adaptive immune response, including T cell and B cell responses, which was maintained up to 5 dpi. These findings may provide a clue to the mechanisms underlying the relatively weak SARS-CoV-2-specific T cell responses and attenuated neutralizing antibody activity observed in asymptomatic or mild COVID-19 patients^55^. Thus, this study provides fundamental information of the in vivo gene expression dynamics of the host immune response as seen in hyper-inflammatory responses elicited by severe SARS-CoV-2 infection in COVID-19 patients^43,56^.

In this study, we suggest that the aged ferrets could be a suitable animal model for the evaluation of new therapeutics against severe COVID-19. Generally, aged individuals have weakened immune function due to the occurrence of immunosenescence, which results in greater susceptibility to viral infections. However, for the same reason, it is hard to keep animals healthy until they reach older ages and as a result, the availability of aged animals is somewhat limited. Thus, the use of immunosuppressed animal models or the development of organoids from aged animals could be of great benefit. However, the lack of relevant immune components in organoid systems would limit systematic infection studies of specific pathogens and impair the investigation of immunological aspects of disease pathogenesis. Thus, comprehensive studies using both in vitro systems and in vivo aged animal models are necessary to understand the pathogenesis of age-associated viral diseases.

Taken together, aged ferrets showed significantly higher virus loads and more severe lung pathology compared to juvenile and young adult ferrets. Moreover, these differences were closely associated with enhanced type I IFN responses and activated M1 macrophages as well as hyper-inflammatory responses in infected aged ferrets. This aged immune-competent ferret model demonstrates for the first time age-dependent pathogenesis of SARS-CoV-2 infection, making it an invaluable animal model to understand the age-dependency of COVID-19 pathogenesis and the detailed underlying mechanism of asymptomatic infection in juveniles and young adults.

## Methods

### Study design for age-dependent pathogenesis in ferrets

SARS-CoV and SARS-CoV-2 antibody-free female ferrets (*Mustela putorius furo*, ID Bio Corporation, Cheongju, Korea) over 36 months (3 years ≤Age, *n* = 9), 12–24 months (1≤ Age ≤2 years, *n* = 9), and under 6 months (Age ≤6 months, *n* = 9) were infected through the intranasal (IN) route with the NMC-nCoV02^28^ strain (GISAID accession number: EPI_ISL_1069194) at a dose of 10^5.8^ TCID_50_ per ferret, while under anesthesia with alfaxalone (2.0 mg/kg) and xylazine (1.0 mg/kg). Following inoculation, body weight and temperature were measured daily and clinical signs of infection such as coughing, rhinorrhea, and reduced activity were noted. Pre-infection values were averaged to obtain a baseline temperature for each ferret. Temperature change was then calculated by subtracting the recorded temperature at each clinical observation time point from the baseline temperature. Nasal washes and fecal specimens were collected every day for 10 days from each group of ferrets to measure viral titers. To measure the infectious live virus in the collected specimens, each sample was inoculated with Vero cells, which were then incubated for 4 days prior to virus isolation. To assess viral replication in various organs of ferrets following SARS-CoV-2 infection, ferrets (*n* = 3/group) were sacrificed at 3 and 5 dpi to harvest their nasal turbinates and lung tissues with individual scissors to avoid cross-contamination. The left lung lobes from the harvested whole lungs were homogenized for virus titration in Vero cells and the right lung lobes were immediately fixed in 10% neutral-buffered formalin solution for further histopathological examinations. For RNA sequencing (RNA-seq) analysis, two different sets of ferrets were used. Briefly, six ferrets per group (juvenile, young adult, and aged) were infected with the same amount of virus as in the virus replication study, and three ferrets from each group were sacrificed at 2 dpi and 5 dpi for RNA-seq analysis of lung homogenates. PBS-treated ferrets (*n* = 9 (Juvenile = 3, young adult = 3, aged = 3)) were intranasally inoculated with phosphate-buffered saline (PBS) and used as the mock-infection group. Mock-infected ferrets (*n* = 9) were euthanized at 2 dpi for RNA-seq analysis of lung homogenates. All animal studies were conducted following protocols approved by the Institutional Animal Care and Use Committee (IACUC) of Chungbuk National University (Approval number CBNUA-1352-20-02).

### Study design for animal-to-animal transmission

To study the animal-to-animal transmission of SARS-CoV-2 by age, we intranasally inoculated SARS-CoV and SARS-CoV-2 antibody-free female ferrets with 10^5.8^ TCID_50_ of SARS-CoV-2. Three ferrets were infected per group (juvenile, young adult, and aged). At 24 h post-infection, SARS-CoV and SARS-CoV-2 antibody-free female naive ferrets (*n* = 3, 12–24 month old) were introduced into direct contact (same cage) with each group of infected ferrets. Clinical signs of infection were assessed and nasal washes and rectal swabs were obtained every day until 11 days post-contact. Blood was collected from contact ferrets to evaluate serum IgG antibody titers at 12 and 21 days post-contact.

### Quantitative real-time RT-PCR (qRT-PCR) to detect SARS-CoV-2 RNA

To measure the viral titer in respiratory and gastrointestinal tracts, nasal washes and rectal swab samples collected from ferrets were suspended in cold phosphate-buffered saline (PBS) containing antibiotics (5% penicillin/streptomycin; Gibco). To measure the viral copy number, total RNA was extracted from the collected samples using RNeasy Mini® kit (QIAGEN, Hilden, Germany) according to the manufacturer’s instructions. A cDNA synthesis kit (Omniscript Reverse Transcriptase; QIAGEN, Hilden, Germany) was used to synthesize single-strand cDNA from total viral RNA. To quantify viral RNA copy number, qRT-PCR was performed for the partial E gene primer set: forward primer, SARS-CoV-2-E-forward, ATGTACTCATTCGTTTCGGAAGAG; and reverse primer, SARS-CoV-2-E-reverse, CTAGAGTTCCTGATCTTCTGGTCTAA with the SYBR Green kit (iQTM SYBR Green supermix kit, Bio-Rad, Hercules, CA, USA). The number of viral RNA copies was calculated and compared to the number of copies of the standard control.

### SARS-CoV-2 isolation

SARS-CoV-2 was isolated from serial nasal wash specimens of each ferret group by inoculating with Vero cells in DMEM (Sigma Aldrich) supplemented with 2% fetal bovine serum (Fisher Scientific), 1 mM L-glutamine (Thermo Fischer), 50 U/mL penicillin (Thermo Fischer), and 50 μg/mL streptomycin (Thermo Fischer). Briefly, specimens were centrifuged at 4 °C at 3,000 × g for 15 min and the supernatants were incubated with Vero cells for 2 h. Media (DMEM) was changed daily and cells were monitored for four days to examine the cytopathic effects (CPEs). To confirm virus isolation, we performed qRT-PCR on supernatants from infected cell cultures using S gene-specific primer sets [Forward (5′-3′): AGGGCAAACTGGAAAGATTGCTGA, Reverse (5′–3′): TCTGTG CAGTTAACATCCTGATAAAGAAC].

### RNA-Sequencing

Total RNA was isolated using TRIzol reagent (Invitrogen) according to the manufacturer’s instructions. RNA quality was assessed with Agilent 2100 Bioanalyzer using an RNA 6000 Nano Chip (Agilent Technologies), and RNA was quantified using an ND-2000 Spectrophotometer (Thermo Fisher Scientific). Extracted RNAs were processed using the TruSeq Stranded mRNA Sample Prep Kit (Illumina) according to the manufacturer’s instructions. High-throughput sequencing was performed as paired-end 150 sequencing runs using NovaSeq 6000 (Illumina). Raw reads were assembled and low-quality reads were filtered using Cutadapt (version 2.8). Filtered reads were aligned on a reference genome downloaded from Ensembl (MusPutFur1.0, Accession number: GCF_000215625.1) using STAR (version 2.7.1a) and annotated with additive human ortholog genes from the human reference database (Biomart database, GRCh38). Gene counts were normalized to valid library size and the dimensional reduction was performed by principal components analysis (PCA) using the top 2 principal components (PCs) throughout the whole samples. Two-sided Wald test was performed to analyze the differentially expressed genes (DEGs) according to each condition with DESeq2 (version 1.26.0)^57^. DEGs in each group were calculated based on age-matched uninfected ferrets, which were determined according to cutoffs of a *p* value <0.05 and a log_2_ fold change >2. To analyze overlapping genes between DEGs identified at 2 and 5 dpi for each age group, the vennCounts and vennDiagram functions from the Limma package (version 3.42.2) were used^58^. To analyze functional profiles of each condition, gene set enrichment analysis (GSEA) was performed in Gene ontology using the Biological process database (GO. BP) and specific public gene set using clusterProfiler (version 3.14.3) and piano (version 2.2.0) packages^59–62^. To compare the gene set enrichment score for the specific gene sets, gene set variation analysis (GSVA) (version 1.34.0) was performed^63^. To obtain the gene sets with significantly upregulated genes from severe COVID-19 patients, we selected the genes based on *p* value <0.05 and log_2_ FC > 2 among the DEGs from the public data sets^42,43^.

### RNAscope in situ hybridization and pathology

SARS-CoV-2 RNA (Spike gene) and ACE2 were detected using the Spike-specific probe and ACE2 probe (Advanced Cell Diagnostics, 848561 and 848151) and visualized using RNAscope 2.5 HD Reagent Kit RED (Advanced Cell Diagnostics, 322360). Lung tissue sections were fixed in 10% neutral-buffered formalin and embedded in paraffin, according to the manufacturer’s instructions, followed by counterstaining with Gill’s hematoxylin #1 (Polysciences, 24242-1000). For pathological examination, the embedded tissues were sectioned and dried for three days at room temperature. Histopathological examination was conducted by hematoxylin and eosin (H&E) staining. Slides were viewed using Olympus IX 71 (Olympus, Tokyo, Japan) microscope with DP controller software to capture images. For statistical analysis of in situ samples, positive foci (most likely resembling single positive cells) were counted manually for each field, and standard deviation was calculated using GraphPad Prism 9 software (Version 9.1.2).

### Histopathology scoring analysis

From 400x magnification area, all positively stained cells (SARS-CoV-2 RNA positive and ACE2 positive) were counted in triplicate and the average was calculated. Lung pathology was graded depending on the degree of inflammation observed in each individual lung at 3 and 5 dpi. All lung tissue slides from infected and mock-infected groups were thoroughly examined and scored with Grades 1 to 4. Scoring was assessed based on the estimated percentage of each lung tissue slide affected by lesions, such as presence of edema, degeneration/necrosis, and infiltrating inflammatory cells in the air passages and lung parenchyma. The lesions that were common to all were considered as background, and thus excluded from the grading scheme. A grade 0 indicates no lesions in the whole tissue slide; Grade 1 indicates 5–20% of the lung tissue slide affected by the lesions; Grade 2 indicates 21–50% of the slide affected by the lesions; Grade 3 indicates 51–80% of the slide affected by the lesions; and Grade 4 indicates 81–100% of the slide affected by the lesions.

### Serologic assay

The neutralizing antibody (NAb) assay against NMC-nCoV02 strain (GISAID accession number: EPI_ISL_1069194) was carried out using a micro-neutralization assay in Vero cells. Collected ferret serum specimens were inactivated at 56 °C for 30 min. Initial 1:2 serum dilutions were made with the medium, and two-fold serial dilutions of all samples were made to a final serum dilution of 1:2 to 1:1024. For each well, 50 μL of serially diluted serum was mixed with 50 μL (equal volume) of 100 TCID_50_ of SARS-CoV-2 and incubated at 37 °C for 1 h to neutralize the infectious virus. The mixtures were then transferred to the Vero cell monolayers. Vero cells were incubated at 37 °C in 5% CO_2_ for four days and monitored for 50% reduction in cytopathic effect (CPE).

### Enzyme-linked immunosorbent assay (ELISA)

Anti-SARS-CoV-2 IgG ELISA assay for ferret serum (FineTest, Wuhan, China, EH4397) targeting Nucleocapsid (N) protein were run according to the manufacturer’s protocol. Serum was diluted 1:50 in each well with the provided sample buffer and incubated at 37 °C for 30 min. Samples were washed three times with a provided wash buffer and incubated with the provided HRP-labeled antibody working solution (0.05 mL/well) at 37 °C for 30 min. After two times of wash, the provided TMB substrate solution was added (0.05 mL/well) and incubated at ambient temperature for 15 min. The provided stop solution was added (0.05 mL/well) and the absorbance was measured at optical density (O.D.) 450 nm with a spectrometer (Bio-Rad; iMark Microplate Reader).

### Ethics statement

All animal experiments were approved by the Medical Research Institute, a member of Laboratory Animal Research Center of Chungbuk National University (LARC) and were conducted in strict accordance and adherence to relevant policies regarding animal handling as mandated under the Guidelines for Animal Use and Care of the Korea Center for Disease Control (K-CDC). Viruses were handled in an enhanced biosafety level 3 (BSL3) containment laboratory as approved by the Korean Centers for Disease Control and Prevention (KCDC-14-3-07).

### Reporting summary

Further information on research design is available in the Nature Research Reporting Summary linked to this article.

## Supplementary information

Supplementary information

Reporting Summary

## Data Availability

The RNA-seq data has been deposited in the GEO database under the primary accession code GSE189619. Source data are provided with this paper.

## Source data

Source Data

## Code availability

All the custom codes used in this study are available at Zenodo (10.5281/zenodo.5708261).
