# Supplementary material for: Age-dependent pathogenic characteristics of SARS-CoV-2 infection in ferrets
Source: Nat Commun. 2022 Jan 10;13:21. doi: 10.1038/s41467-021-27717-3 (PMC8748994; doi:10.1038/s41467-021-27717-3)
Supplement: Supplementary file 1 — Supplementary information [file 41467_2021_27717_MOESM1_ESM.pdf]

Supplementary information for

Age-dependent pathogenic characteristics of SARS-CoV-2 infection in ferrets

Young-Il Kim[1,2,6†], Kwang-Min Yu[1,2†], June-Young Koh[3†], Eun-Ha Kim[1,2], Se-Mi Kim[1,2,6], Eun Ji Kim[1,2], Mark Anthony B. Casel[1,2], Rare Rollon[1], Seung-Gyu Jang[1], Min-Suk Song[1,2], Su-Jin Park[4], Hye Won Jeong[1], Eung-Gook Kim[1], Ok-Jun Lee[1], Yong-Dae Kim[1], Younho Choi[5], Shin-Ae Lee[5], Youn Jung Choi[5], Su-Hyung Park[3], Jae U. Jung[5]*, and Young Ki Choi[1,2,6]*

[1]College of Medicine and Medical Research Institute, Chungbuk National University, Cheongju, Republic of Korea.
[2]Zoonotic Infectious Diseases Research Center, Chungbuk National University, Cheongju, Korea.
[3]Graduate School of Medical Science and Engineering, Korea Advanced Institute of Science and Technology (KAIST), Daejeon, Republic of Korea.
[4]Division of Life Science, Research Institute of Life Science, Gyeongsang National University, Jinju 52828, Korea.
[5]Cancer Biology Department and Global Center for Pathogens Research and Human Health, Lerner Research Institute, Cleveland Clinic, Cleveland, Ohio, USA.
[6]Center for Study of Emerging and Re-emerging Viruses, Korea Virus Research Institute, Institute for Basic Science (IBS), Daejeon 34126, Republic of Korea

[†]These authors contributed equally

*Corresponding authors: Jae U. Jung and Young Ki Choi

Email: jungj@ccf.org, E-mail: choiki55@chungbuk.ac.kr or choiki55@ibs.re.kr

Supplementary Figure 1 – 7

Supplementary Table 1 – 4

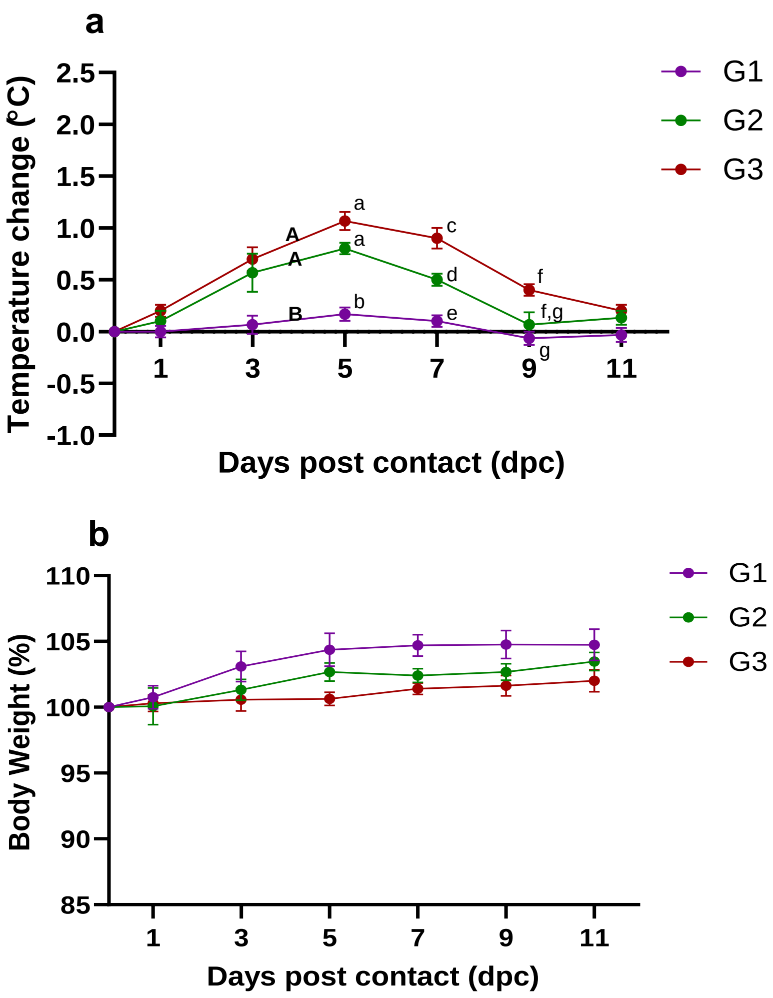

Supplementary Fig. 1. Change in body temperature and weight of direct contact ferrets. Temperature changes (a) and relative body weight (b) were measured in direct contact transmission ferrets from each different age group. Temperature is represented as °C and weight is demonstrated as a percentage of the initial body weight. Data are presented as mean ± SEM (n=3/group). Groups with the same letter are of the same subgroup in the post-hoc analysis. Lower case letters indicate significant differences at each time point (*P* values are a vs b: 0.0024 or 0.00035, c vs d: 0.02707, c vs e: 0.00082, d vs e: 0.02707, f vs g: 0.026 (a)), while upper case letters indicate significant differences of area under the curve as change over the entire period (*P* values are A vs B: 0.01162 or 0.00068). The area under the curve was calculated using Kruskal–Wallis with Bonferroni as a post-hoc analysis. Data were analyzed in GraphPad Prism 9.1.2. Source data are provided as a Source Data file.

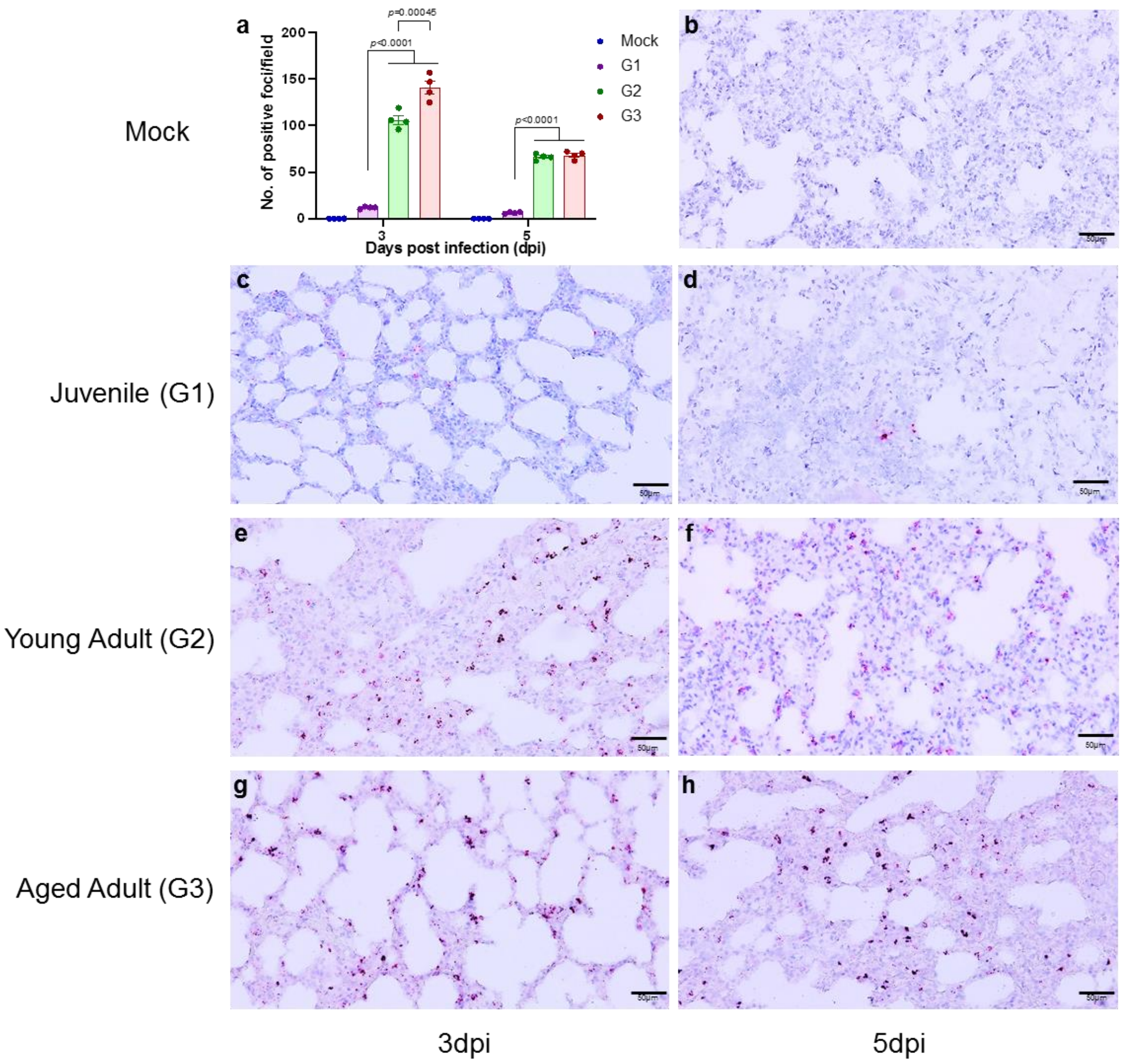

Supplementary Fig. 2. *In situ* lung analysis and histopathology of SARS-CoV-2 infected ferret groups. Ferrets were inoculated with $10^{5.8}$ $TCID_{50}$ of NMC-nCoV02 virus. Lung tissues were harvested on days 3 (c, e, and g) (n=3/group) and 5 (d, f, and h) (n=3/group) post-inoculation. Lung regions were compared by histopathology among the different age groups of ferrets: (a) Quantification of SARS-CoV-2 RNA-positive foci in lung sections; four fields were counted from section (n=6/group). Data are presented as mean values ± SEM. Statistical analysis was calculated using Kruskal–Wallis with Bonferroni as a post-hoc analysis. Data were analyzed in GraphPad Prism 9.1.2. RNA-positive cells in lung tissues of Mock infected (b), juveniles (less than 6 months, G1 group) (c and d), young adults (1 to 2 years, G2 group) (e and f), and aged ferrets (older than 3 years) (g and h). Magnification 200x and scale bar 50 µm. Source data are provided as a Source Data file.

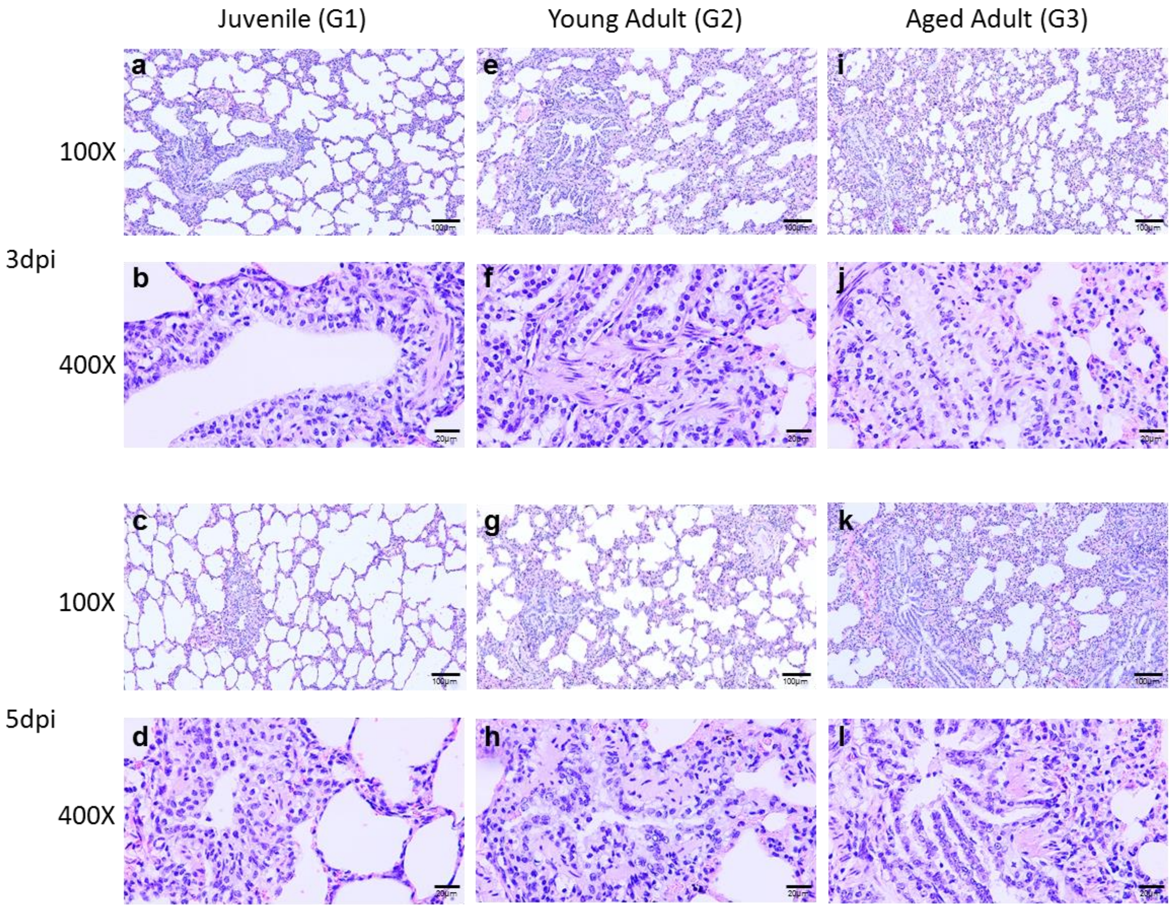

Supplementary Fig. 3. Lung histopathology of SARS-CoV-2 infected ferret groups. Ferrets were inoculated with $10^{5.8}$ $TCID_{50}$ of NMC-nCoV02 virus. Lung tissues were harvested on days 3 and 5 post-inoculation (n=6 per group). Histopathological lung regions were compared among the different age groups of ferrets: (a-d) juvenile (less than 6 months, G1 group), (e-h) young adults (1 to 2 years, G2 group), and (i-l) aged ferrets (older than 3 years). Histopathological observations indicated that moderate interstitial pneumonia with thickened alveolar septa (a, c, e, g, i, and k, magnification 100x and scale bar 100μm). G3 group showed more severe lung damage and infiltration of lymphocytes compared G1 and G3 groups (b, d, f, h, j, and l, magnification 400x and scale bar 20μm).

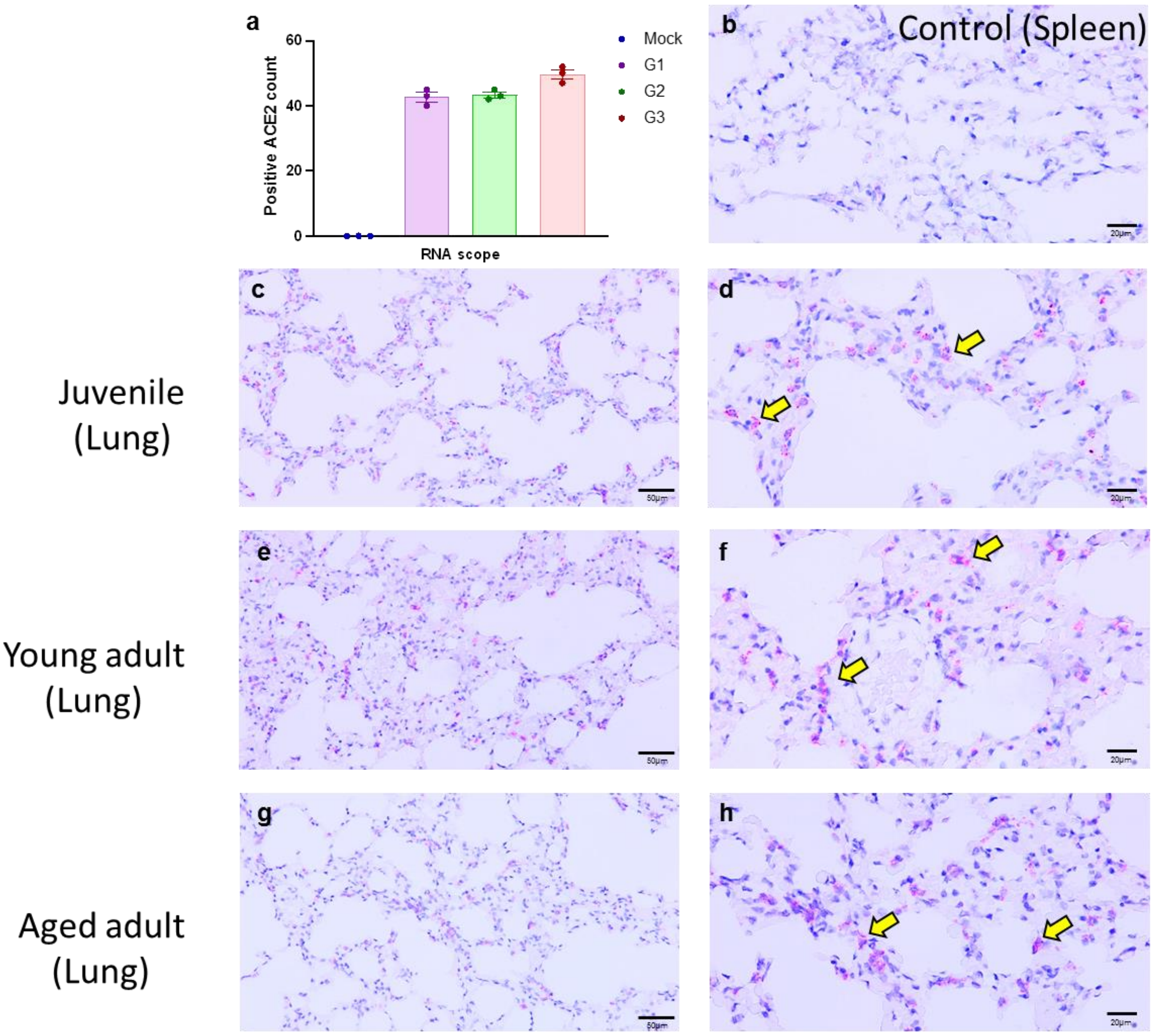

Supplementary Fig. 4. RNAscope in situ hybridization of ferret ACE2 receptor expression in the lung. To quantitate ACE2 expression in ferret lungs by age group (n=3 per group), RNAscope in situ hybridization was performed using an ACE2 probe and visualized using RNAscope 2.5 HD Reagent Kit RED. Positive ACE2 cumulative number from ACE2 stained lung cells in each slide (400x magnification) and data are presented as mean values ± SEM (n=3 per group) (a). Spleen tissue section as ACE2 negative control (b), ACE2-positive cells (Yellow arrows) in lung tissues of juvenile (≤ 6 months, G1 group) (c and d), young adult (1 ≤ age ≤ 2 years, G2 group) (e and f), and aged ferrets (3-year ≤ ages) (g and h). Magnification 200x and scale bar 50μm (a, c, e, and g). Magnification 400x and scale bar 20μm (b, d, f, and h). Statistical analysis was calculated using Kruskal–Wallis with Bonferroni as a post-hoc analysis. Data were analyzed in GraphPad Prism 9.1.2. Source data are provided as a Source Data file.

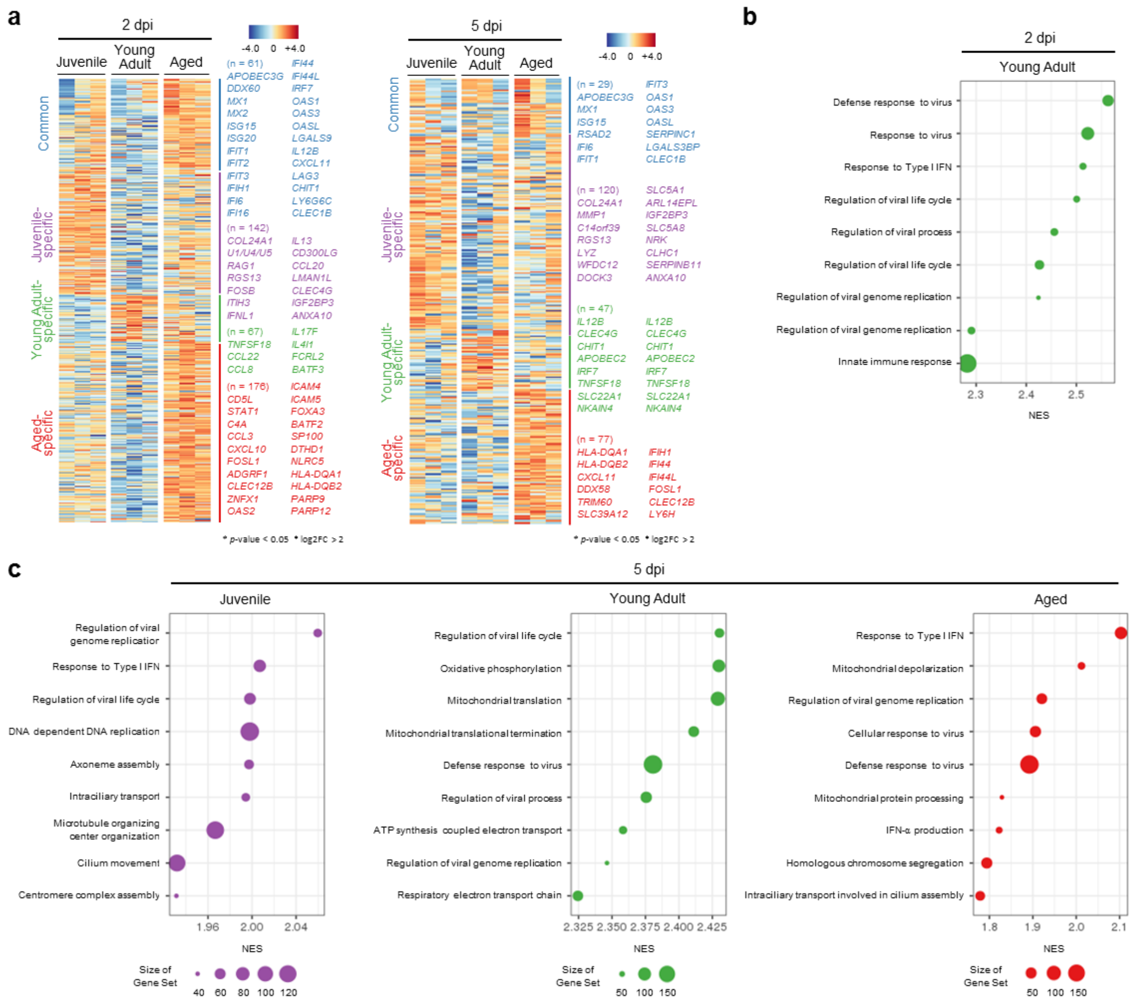

Supplementary Fig. 5. Differentially expressed genes in comparison among juvenile, young adult, and aged groups at 2 and 5 dpi. Heatmap of age-specific differentially expressed genes (DEGs) compared to age-matched control ferrets at 2 and 5 dpi (a). Heatmap colors from red to blue represent low to high enrichment, respectively. While changes of gene expression are conveyed using a color gradation (red, light red, light blue and blue), and unchanged expression is represented by white color. Two-sided Wald test was performed to analyze the differentially expressed genes (DEGs). Plots with normalized enrichment score (NES) from enrichment analysis of representative Gene Ontology (GO) biological pathway in young adult ferrets at 2 dpi (b). Plots with NES from enrichment analysis of representative GO biological pathway in juvenile, young adult, and aged ferrets at 5 dpi (c).

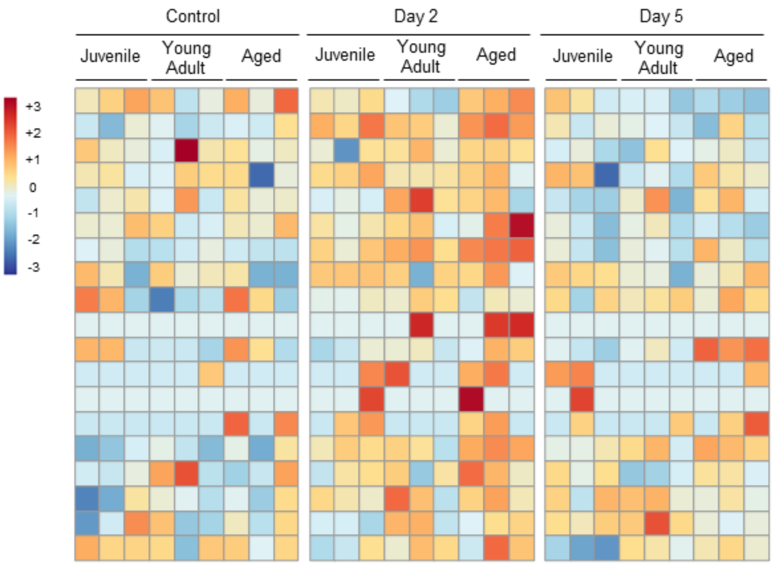

	P-value		
	control vs D2	control vs D5	D2 vs D5
IL1B		0.0092	0.08
TNF	< 0.0001		0.0001
IL6			0.05
CXCL8	0.09		
CCL2			
CCL4		0.0052	0.0064
CXCL10	< 0.0001		0.0005
CSF3			
CSF2			
IFNB1	0.06		0.06
IFNG			
IL17A	0.06		
IL4			
IL2			
IL7	0.0007	0.0014	
IL12A			
IL18	0.0095	0.0201	
IL10		0.1	
IL1RN		0.08	

Supplementary Fig. 6. Expression of various cytokines and chemokines in the lung of SARS-CoV-2-infected ferrets. The heatmap shows the induction of cytokines and chemokines as measured by RNA sequencing in aged, juvenile, and young adult ferrets at two time points (2 and 5 dpi). The color gradient represents gene expression levels. Two-sided unpaired t-test was performed to analyze the normalized gene expressions. *P*-value was calculated statistical significance compared with Mann-Whitney U tests.

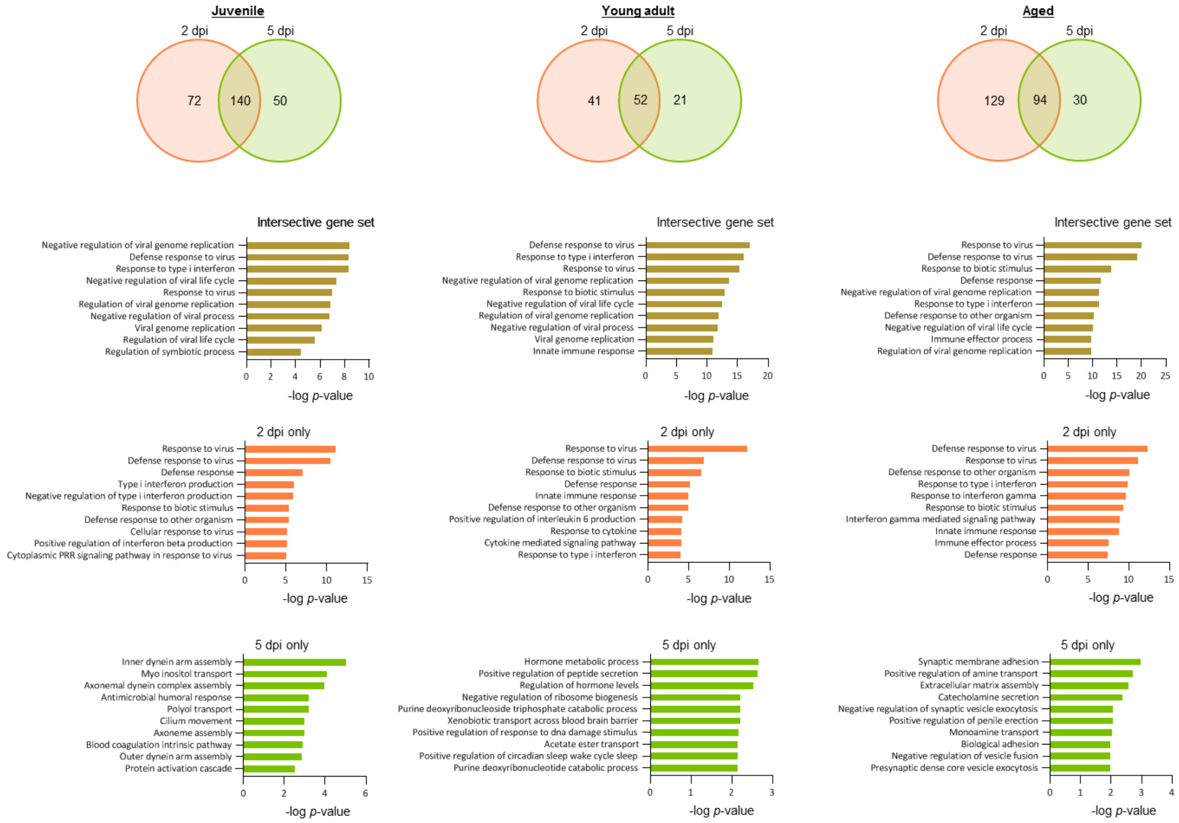

Supplementary Fig. 7. Functional enrichment analysis of inflammatory immune response-associated genes in SARS-CoV-2-infected ferrets. Venn diagram showing the intersection of the gene sets that are differently upregulated in each SARS-CoV-2-infected ferret groups between 2 dpi vs. 5 dpi. Bar plots represent overlapping gene sets that are highly upregulated at 2 dpi and/or 5 dpi.

Supplementary Table 1. Clinical scores of individual ferrets infected with SARS-CoV-2

Group		0dpi	2dpi	4dpi	6dpi	8dpi	10dpi
	Cough	0.00	0.00	0.00	0.00	0.00	0.00
	Runny nose	0.00	0.00	0.00	0.00	0.00	0.00
	Movement, activity	0.00	0.00	0.00	0.00	0.00	0.00
	Cough	0.00	0.00	0.00	0.00	0.00	0.00
	Runny nose	0.00	0.00	1.00	0.00	0.00	0.00
	Movement, activity	0.00	0.00	0.00	0.00	0.00	0.00
	Cough	0.00	0.00	0.00	0.00		
	Runny nose	0.00	1.00	0.00	0.00		
	Movement, activity	0.00	0.00	0.00	0.00		
	Cough	0.00	0.00	0.00	0.00		
	Runny nose	0.00	1.00	0.00	0.00		
	Movement, activity	0.00	0.00	0.00	0.00		
	Cough	0.00	0.00				
**G1 (Juvenile)**	Runny nose	0.00	1.00				
	Movement, activity	0.00	1.00				
	Cough	0.00	0.00	0.00	0.00		
	Runny nose	0.00	0.00	0.00	0.00		
	Movement, activity	0.00	0.00	1.00	0.00		
	Cough	0.00	0.00				
	Runny nose	0.00	1.00				
	Movement, activity	0.00	1.00				
	Cough	0.00	0.00	0.00	0.00	0.00	0.00
	Runny nose	0.00	0.00	1.00	0.00	0.00	0.00
	Movement, activity	0.00	1.00	0.00	0.00	0.00	0.00
	Cough	0.00	0.00				
	Runny nose	0.00	1.00				
	Movement, activity	0.00	1.00				
**Average**	Cough	0.00	0.00	0.00	0.00	0.00	0.00
	Runny nose	0.00	0.56±0.50	0.33±0.47	0.00	0.00	0.00

	Movement, activity	0.00	0.44±0.50	0.17±0.37	0.00	0.00	0.00
	Cough	0.00	0.00				
	Runny nose	0.00	1.00				
	Movement, activity	0.00	1.00				
	Cough	0.00	1.00	1.00	1.00		
	Runny nose	0.00	0.00	2.00	2.00		
	Movement, activity	0.00	0.00	2.00	2.00		
	Cough	0.00	1.00				
	Runny nose	0.00	0.00				
	Movement, activity	0.00	1.00				
	Cough	0.00	1.00				
	Runny nose	0.00	1.00				
	Movement, activity	0.00	2.00				
**G2 (Young adult)**	Cough	0.00	0.00	1.00	1.00		
	Runny nose	0.00	0.00	1.00	1.00		
	Movement, activity	0.00	1.00	2.00	2.00		
	Cough	0.00	0.00	1.00	0.00		
	Runny nose	0.00	0.00	1.00	1.00		
	Movement, activity	0.00	1.00	2.00	1.00		
	Cough	0.00	0.00	1.00	1.00	0.00	0.00
	Runny nose	0.00	0.00	2.00	1.00	0.00	0.00
	Movement, activity	0.00	0.00	1.00	2.00	1.00	0.00
	Cough	0.00	0.00	1.00	1.00	0.00	0.00
	Runny nose	0.00	0.00	1.00	1.00	0.00	0.00
	Movement, activity	0.00	0.00	2.00	2.00	1.00	0.00
	Cough	0.00	0.00	1.00	1.00	0.00	0.00
	Runny nose	0.00	0.00	1.00	1.00	0.00	0.00
	Movement, activity	0.00	0.00	2.00	2.00	1.00	0.00
**Average**	Cough	0.00	0.33±0.47	1.00	0.83±0.37	0.00	0.00
	Runny nose	0.00	0.22±0.42	1.33±0.47	1.17±0.37	0.00	0.00
	Movement, activity	0.00	0.67±0.67	1.83±0.37	1.83±0.37	1.00	0.00
**G3**	Cough	0.00	1.00	1.00	1.00	0.00	0.00

**(Aged adult)**	Runny nose	0.00	0.00	2.00	2.00	1.00	0.00
	Movement, activity	0.00	0.00	2.00	1.00	1.00	0.00
	Cough	0.00	1.00	1.00	1.00		
	Runny nose	0.00	0.00	2.00	1.00		
	Movement, activity	0.00	1.00	2.00	2.00		
	Cough	0.00	0.00	1.00	1.00		
	Runny nose	0.00	0.00	2.00	1.00		
	Movement, activity	0.00	0.00	2.00	2.00		
	Cough	0.00	1.00				
	Runny nose	0.00	1.00				
	Movement, activity	0.00	2.00				
	Cough	0.00	1.00				
	Runny nose	0.00	1.00				
	Movement, activity	0.00	2.00				
	Cough	0.00	0.00	1.00	1.00		
	Runny nose	0.00	0.00	1.00	1.00		
	Movement, activity	0.00	0.00	2.00	2.00		
	Cough	0.00	0.00	1.00	1.00	0.00	0.00
	Runny nose	0.00	0.00	2.00	1.00	0.00	0.00
	Movement, activity	0.00	0.00	2.00	2.00	1.00	1.00
	Cough	0.00	0.00	1.00	1.00	0.00	0.00
	Runny nose	0.00	0.00	1.00	1.00	1.00	1.00
	Movement, activity	0.00	0.00	2.00	2.00	1.00	1.00
	Cough	0.00	1.00				
	Runny nose	0.00	2.00				
	Movement, activity	0.00	2.00				
	Cough	0.00	0.56±0.50	1.00	1.00	0.00	0.00
**Average**	Runny nose	0.00	0.44±0.68	1.67±0.47	1.17±0.37	0.67±0.47	0.33±0.47
	Movement, activity	0.00	0.78±0.92	2.00	1.83±0.37	1.00	0.67±0.47

Observational clinical symptoms: Cough, rhinorrhea, movement, and activity.

Score: 0; normal, 1: occasional, mild reduced activity, 2: frequent, reduced activity.

Scores were measured by observation of clinical symptoms for at least 20 minutes in each group of ferrets based on the following criteria: Cough: 0; no evidence of cough, 1; occasional cough, 2; frequent cough (score 2).

Rhinorrhea: 0; no nasal rattling or sneezing, 1; moderate nasal discharge on external nares, 2; severe nasal discharge on external nares.

Movement, activity: 0; normal movement and activity, 1; mild reduced movement and activity, 2; evidence of reduced movement and activity.

Supplementary Table 2. Clinical symptom score in contact group ferrets.

Group		0dpc	1dpc	3dpc	5dpc	7dpc	9dpc	11dpc
	Cough	0.00	0.00	0.00	0.00	0.00	0.00	0.00
	Runny nose	0.00	0.00	0.00	0.00	0.00	0.00	0.00
	Movement, activity	0.00	0.00	0.00	0.00	0.00	0.00	0.00
	Cough	0.00	0.00	0.00	0.00	0.00	0.00	0.00
**G1 (Juvenile)**	Runny nose	0.00	0.00	0.00	0.00	0.00	0.00	0.00
	Movement, activity	0.00	0.00	0.00	0.00	0.00	0.00	0.00
	Cough	0.00	0.00	0.00	0.00	0.00	0.00	0.00
	Runny nose	0.00	0.00	0.00	0.00	0.00	0.00	0.00
	Movement, activity	0.00	0.00	0.00	0.00	0.00	0.00	0.00
	Cough	0.00	0.00	0.00	0.00	0.00	0.00	0.00
**Average**	Runny nose	0.00	0.00	0.00	0.00	0.00	0.00	0.00
	Movement, activity	0.00	0.00	0.00	0.00	0.00	0.00	0.00
	Cough	0.00	0.00	0.00	0.00	0.00	0.00	0.00
	Runny nose	0.00	0.00	1.00	1.00	0.00	0.00	0.00
	Movement, activity	0.00	0.00	1.00	0.00	0.00	0.00	0.00
	Cough	0.00	0.00	0.00	0.00	0.00	0.00	0.00
**G2 (Young adult)**	Runny nose	0.00	0.00	1.00	0.00	0.00	0.00	0.00
	Movement, activity	0.00	0.00	1.00	1.00	0.00	0.00	0.00
	Cough	0.00	0.00	0.00	0.00	0.00	0.00	0.00
	Runny nose	0.00	0.00	0.00	0.00	0.00	0.00	0.00
	Movement, activity	0.00	0.00	1.00	0.00	0.00	0.00	0.00
	Cough	0.00	0.00	0.00	0.00	0.00	0.00	0.00
**Average**	Runny nose	0.00	0.00	0.67±0.47	0.33±0.47	0.00	0.00	0.00
	Movement, activity	0.00	0.00	1.00	0.33±0.47	0.00	0.00	0.00
	Cough	0.00	0.00	0.00	0.00	0.00	0.00	0.00
	Runny nose	0.00	0.00	1.00	1.00	1.00	0.00	0.00
**G3 (Aged adult)**	Movement, activity	0.00	0.00	1.00	2.00	1.00	0.00	0.00
	Cough	0.00	0.00	0.00	0.00	0.00	0.00	0.00
	Runny nose	0.00	0.00	1.00	1.00	0.00	0.00	0.00
	Movement, activity	0.00	0.00	1.00	1.00	1.00	0.00	0.00

	Cough	0.00	0.00	0.00	0.00	0.00	0.00	0.00
	Runny nose	0.00	0.00	1.00	2.00	1.00	0.00	0.00
	Movement, activity	0.00	0.00	1.00	1.00	1.00	0.00	0.00
**Average**	Cough	0.00	0.00	0.00	0.00	0.00	0.00	0.00
	Runny nose	0.00	0.00	1.00	1.33±0.47	0.67±0.47	0.00	0.00
	Movement, activity	0.00	0.00	1.00	1.33±0.47	1.00	0.00	0.00

Observational clinical symptoms: Cough, rhinorrhea, movement, and activity.

Score: 0; normal, 1: occasional, mild reduced activity, 2: frequent, reduced activity.

Scores were measured by observation of clinical symptoms for at least 20 minutes in each group of ferrets based on the following criteria: Cough: 0; no evidence of cough, 1; occasional cough, 2; frequent cough (score 2).

Rhinorrhea: 0; no nasal rattling or sneezing, 1; moderate nasal discharge on external nares, 2; severe nasal discharge on external nares.

Movement, activity: 0; normal movement and activity, 1; mild reduced movement and activity, 2; evidence of reduced movement and activity.

Supplementary Table 3. Genes used in principal component analysis (PCA)

PC1	PC2
CLCA1	*RSAD2*
DNAH9	*ISG15*
MUC5AC	*MX1*
BPIFB1	*IFIT3*
HBB	*CMPK2*
CDHR4	*DHX58*
ERICH3	*OAS3*
CFAP43	*IFI6*
ECT2L	*USP18*
HBA2	*MX2*
DNAH5	*OAS1*
LPO	*OASL*
DNAH10	*APOBEC3G*
CFAP65	*HERC6*
DNAH1	*UBA7*
ERN2	*DDX58*
TMC5	*IFIT2*
VMO1	*IRF7*
CAPS	*RNF213*
MAPK15	*DDX60*
AGR2	*SAMD9L*
RSPH1	*GBP1*
EML6	*IFIH1*
GP2	*HERC5*
BPIFA1	*NLRC5*
LAYN	*CXCL10*
CPA3	IFI44
DRC7	CXCL11
FOXJ1	IFI44L

DNAH11	SAMHD1
DNAI2	PARP14
DNAH7	BATF2
SPAG6	LGALS9C
ACE	UBE2L6
HYDIN	TRANK1
MUC13	STAT1
CCDC114	TRIM6-TRIM34
TFF3	EPSTI1
AK7	DTX3L
SCGB3A1	C3
CCDC180	LGALS3BP
CFAP52	TRIM22
LRRC43	ZNFX1
TTLL10	BST2
NME9	PYHIN1
TTC21A	C4A
VWA3A	NT5C3A
CCDC146	PARP12
STMND1	PARP15
CCDC40	ZBP1
UBXN10	OAS2
WDR63	EIF2AK2
CFAP45	PML
COL15A1	ETV7
FAM216B	NUP153
DNAAF1	AEBP1
SLC44A4	HSH2D
C17orf97	HELZ2
WDR38	PARP9
COL1A1	IL4I1

CSF3R	IFIT5
DNAH6	HLA-DQA1
VWA3B	PARM1
CAPSL	AC104389.5
WDR66	PRG4
ANKMY1	STAT2
CFAP46	C19orf66
PLEKHS1	IGHA2
ZCCHC18	APOL5
SNTN	CHIT1
CFAP126	SIGLEC1
CDHR3	SP100
APOA1	PLSCR1
FRMPD2	TRIM25
ALAS2	ERAP2
DCDC2	TDRD7
ACOD1	RTP4
TMEM2	
COL3A1	
CFAP99	
FAM183A	
CATIP	
CFAP157	
LRRC23	
TOGARAM2	
ALS2CR12	
PTN	
AC010255.3	
MAP3K19	
THBD	

Supplementary Table 4. A list of differentially expressed genes (DEGs)

[at 2 dpi]

Common	Juvenile	Young Adult	Aged
ZBP1	*CCNJL*	*RARRES1*	*CA10*
MX1	*EPHA5*	*ESCO2*	*DNTT*
RSAD2	*BTNL9*	*CCL22*	*MUC5AC*
MX2	*CAV3*	*DDIAS*	*ISLR2*
EBF2	*GPX6*	*TMEM255B*	*KIAA1024*
BTNL2	*OSM*	*WNT5B*	*TTLL8*
ACOD1	*SORCS1*	*C10ORF99*	*BST2*
SHISA3	*LYZ*	*PAX5*	*ZNFX1*
TMEM81	*L1CAM*	*RBP5*	*FSHR*
LGALS9C	*SLC5A7*	*TMIGD2*	*TLR7*
PML	*GLTPD2*	*SIGLEC1*	*CLEC4F*
CXCL10	*EEF1A2*	*PAX8*	*MS4A2*
IFI44L	*COL3A1*	*ESPL1*	*HIST1H4E*
SLFN14	*LRIT3*	*LGSN*	*RIPOR3*
CXCL11	*MGAM2*	*FXYD2*	*TENM2*
MYL7	*CILP*	*LY6E*	*ST6GALNAC1*
FAP	*COL5A3*	*CDK1*	*CSF3R*
CLEC4G	*APLNR*	*MXRA5*	*CRYBG2*
CD177	*TFF2*	*KCNK2*	*C3AR1*
TELOMERASE-VERT	*MYH6*	*SCG3*	*TRAV21*
TUBB1	*SNAP25*	*LY6H*	*KRT24*
TRANK1	*IGHV7-4-1*	*ACOT6*	*VWCE*
NUGGC	*ITIH3*	*CDKN3*	*NLRC5*
TNF	*TNFAIP6*	*HIST1H4G*	*EPX*
IFI6	*NRK*	*SHOX2*	*XKRX*
SAMD9L	*LMOD2*	*SKA1*	*ACP7*
UBE2L6	*KRT23*	*CRABP2*	*S100A12*
ASB16	*ATP1A2*	*SECTM1*	*BATF2*
HERC6	*ADAMTS17*	*NOXO1*	*PIANP*
HERC5	*COMP*	*NCAPG*	*TTC36*
CAPN9	*CSRP3*	*C12ORF40*	*EN1*

LY6G6C	*ZSCAN10*	*C4A*	*KIAA1683*
GBP1	*DHRS7C*	*TRIM9*	*LMTK3*
HES4	*UCP3*	*CREB3L1*	*OMG*
EPSTI1	*ADORA1*	*TCTEX1D1*	*KIR3DL3*
CPA3	*HMCN2*	*LBP*	*GPR142*
FER1L6	*AL662899.1*	*ADAD1*	*CHIT1*
APOBEC3G	*CD300LG*	*OTX2*	*TREML1*
DDX58	*C10ORF90*	*MB21D1*	*ADGRF1*
LVRN	*ST18*	*NOTO*	*TCN1*
EPHX4	*SYT4*	*CPA4*	*PADI4*
ISG15	*CPA1*	*BATF3*	*IL17F*
PARP15	*TNNC1*	*GZMA*	*CDH17*
IFI44	*PLA2G4D*	*LY86*	*LTF*
PARP14	*TDRD9*	*TVP23A*	*RHAG*
BPIFB1	*ANK1*	*HTR3A*	*CHST4*
WBSCR17	*GJB1*	*SERPINB7*	*CEBPE*
NT5C3A	*PPP1R1A*	*XKR9*	*ELFN1*
CRISP3	*PLP1*	*PARP12*	*RAB44*
PARM1	*PADI1*	*ISG20*	*IL12B*
LGALS3BP	*SERPINB11*	*SPC24*	*RAG2*
IL5RA	*SLC22A1*	*APOC1*	
IFIT5	*COL15A1*	*CD8B*	
LPO	*CD1C*	*GLRB*	
IFIT2	*RLBP1*	*MAP7D2*	
GATA1	*THBS4*	*DNASE1L3*	
IFIT3	*SLC6A5*	*MXD3*	
DDX60	*NPPC*	*SPC25*	
RNF213	*EGFLAM*	*AC104389.5*	
HELZ2	*MMP27*	*ANKRD33B*	
BPIFA1	*CHSY3*	*GRIA2*	
TDO2	*NPPA*	*COL19A1*	
CLCA1	*UGT1A6*	*RTP4*	
RAG1	*CYTL1*	*TRIM71*	
MMP8	*MYOZ2*	*LRR1*	
DTX3L	*CSMD2*	*CDCA8*	

EIF2AK2	*GABRD*	*PKMYT1*
PYHIN1	*UGT1A8*	*GPR83*
OAS1	*GPIHBP1*	*TMEM221*
MMP9	*TCP11*	*IL4I1*
ETV7		*LIPG*
CYP26A1		*CCNB3*
OAS2		*C5*
OAS3		*PLAC8*
IRF7		*CCL8*
TFF1		*GJA3*
UBA7		*PI3*
DUOX2		*TREX1*
OTOG		*OIP5*
CPXM1		*HOXC9*
TRIM6-TRIM34		
CAMP		
NTRK1		
STAT1		
TIMD4		
GPR31		
C1QTNF3-AMACR		
PARP9		
5S_RRNA_23163		
NLRP14		
F9		
HSH2D		
CMPK2		
SERPINC1		
HDC		
CD5L		
USP18		
OASL		
SAMHD1		
IFIH1		
CLEC1B		

DHX58			
AGR2			
TRIM22			

[at 5 dpi]

Common	Juvenile	Young Adult	Aged
LUM	*EPHA7*	*ZBP1*	*RARRES1*
MMP8	*MMP1*	*IL10*	*CA10*
MX1	*GPX6*	*CD70*	*CCL22*
TMEM255B	*TM4SF4*	*MACC1*	*CNTN6*
DTX3L	*SORCS1*	*PAX5*	*KCNIP3*
EIF2AK2	*LYZ*	*MYBPC1*	*AMBN*
RSAD2	*L1CAM*	*PLXDC1*	*ETV7*
WNT5B	*EMCN*	*ETNPPL*	*SFRP5*
MX2	*AZGP1*	*LGALS9C*	*CYP26A1*
PYHIN1	*COL3A1*	*DISP2*	*LRFN5*
KERA	*MFAP2*	*RBP7*	*MANSC4*
SHISA3	*SCN10A*	*DPM3*	*IFNG*
OAS1	*ELF5*	*TMIGD2*	*CD207*
MMP9	*LIX1*	*LGALS12*	*ATP13A5*
IFI44L	*COL5A3*	*SIGLEC1*	*MKX*
MYL7	*TFF2*	*CILP*	*PDCD1*
OAS3	*ACOT1*	*FXYD2*	*CD69*
FAP	*DSG3*	*LY6E*	*LY6H*
MYL4	*TFF1*	*MELTF*	*VIP*
TFF3	*MS4A2*	*ATP13A4*	*KANK4*
ARPP21	*SNAP25*	*THRSP*	*AC090227.1*
SCG2	*ST6GALNAC1*	*COX20*	*LNP1*
MXRA5	*TNFAIP6*	*ACOT6*	*CRYBG2*
KCNK2	*NRK*	*S100A9*	*ANKRD35*
SCG3	*HEPACAM2*	*S100A8*	*CD80*
CLEC4G	*TRANK1*	*VIT*	*NOXO1*
C17ORF78	*KRT23*	*RAMP3*	*C4BPB*
CD177	*MPO*	*RBPJL*	*HTR2B*

TELOMERASE-VERT	*CXCL14*	*NYAP2*	*THY1*
RETNLB	*SNX31*	*DGKB*	*UBE2L6*
UBA7	*EPX*	*HAAO*	*C4A*
AC140504.1	*ADCY8*	*UCP2*	*SLC5A5*
IFI6	*ADAMTS12*	*JCHAIN*	*CLGN*
SAMD9L	*HAPLN3*	*OC90*	*SLC22A13*
HERC6	*ADAMTS17*	*SLC7A4*	*PROKR1*
HERC5	*CAPN9*	*NEURL1B*	*THPO*
CA4	*MDK*	*DUOX2*	*FLRT1*
FAM196B	*TNN*	*MAP1LC3C*	*C10ORF10*
TRIM6-TRIM34	*HAS2*	*CA6*	*GBP1*
PCK1	*CD300LB*	*MALRD1*	*NXPH2*
EPSTI1	*SLC26A10*	*SPP1*	*TINAG*
CAMP	*FCER1A*	*S100A12*	*SLC25A48*
GPR158	*CPXM1*	*PLK5*	*CTBS*
APOBEC3G	*OTX2*	*BATF3*	*CCDC30*
DDX58	*GPC5*	*P2RY12*	*GPR39*
TIMD4	*CPA3*	*CDKN2A*	*RIC3*
ISG15	*ST18*	*LRRC75B*	*IL33*
PARP15	*CUBN*	*PVRIG*	*LGI1*
IFI44	*KCNK13*	*GZMA*	*CGREF1*
PARP9	*LVRN*	*EPHX4*	*TMPRSS11A*
PARP14	*PLA2G4D*	*XKR9*	*SCD5*
BPIFB1	*DDX11*	*CHIT1*	*PPBP*
PARP12	*ANXA10*	*SULT2B1*	*ADGRF1*
NLRP14	*SHCBP1*	*DHRS9*	*TCN1*
NA	*RFLNB*	*APOC1*	*STAC2*
NT5C3A	*GJB1*	*COL8A1*	*ACSBG1*
GAP43	*F9*	*PADI4*	*CDH16*
HSH2D	*RIT2*	*DNASE1L3*	*AC104389.5*
KLHDC8A	*KIT*	*MATN4*	*IL7R*
CMPK2	*GLRB*	*SLC22A1*	*GRIA2*
PCOLCE2	*CD226*	*CYGB*	*GSG1L2*
LTF	*PLCB1*	*CYSRT1*	*COL19A1*
ZNF215	*PADI1*	*RIMS3*	*C1R*

FGF13	*SERPINB11*	*RAB42*	*SERPINC1*
SPC25	*LY6L*	*PLAC1*	*DOC2A*
CRISP3	*CFI*	*PWWP2B*	*C1S*
CCL13	*PCDH12*	*TREX1*	*SEMA3E*
RTP4	*COL15A1*	*MUC15*	*PLA2G7*
LGALS3BP	*COL11A2*	*GYG2*	*LYPD6*
IFIT5	*HDC*	*NKAIN1*	*GPR83*
ABCA13	*IL5RA*		*SAMHD1*
IFIT2	*LPO*		*NDST3*
USP18	*C1ORF54*		*CLEC1B*
OASL	*CD1C*		*PLAC8*
IFIT3	*PCDH17*		*RGS1*
DDX60	*RNF213*		*KCNMB2*
IFIH1	*ADAMTS6*		*PTCHD1*
BPIFA1	*THBD*		*AP1S3*
DHX58	*EDNRB*		*ENPP3*
PI3	*CHSY3*		*ANKRD55*
PGLYRP1	*C1QTNF6*		*OLR1*
TRIM22	*TESPA1*		
	PDGFD		
	UGT1A6		
	SLITRK6		
	CLCA1		
	NOS2		
	TRPM6		
	EPHB2		
	AHSP		
	GABRD		
